# Response analysis of *Pinus sibirica* to pine wood nematode infection through transcriptomics and metabolomics study

**DOI:** 10.3389/fpls.2024.1383018

**Published:** 2024-05-07

**Authors:** Jiawei Zhang, Lingfang Ye, Qiaoli Chen, Feng Wang

**Affiliations:** ^1^ Key Laboratory of Alien Forest Pests Detection and Control-Heilongjiang Province, College of Forestry, Northeast Forestry University, Harbin, China; ^2^ Key Laboratory of Sustainable Forest Ecosystem Management-Ministry of Education, Northeast Forestry University, Harbin, China; ^3^ State Key Laboratory of Tree Genetics and Breeding, College of Forestry, Northeast Forestry University, Harbin, Heilongjiang, China; ^4^ Key Laboratory of Nation Forestry and Grassland Administration on Northeast Area Forest and Grass Dangerous Pest Management and Control, Shenyang Institute of Technology, Fushun, Liaoning, China

**Keywords:** *Bursaphelenchus xylophilus*, pine wilt disease, *Pinus sibirica*, transcriptome, metabolome, multi-omics

## Abstract

*Pinus sibirica* is primarily distributed in Siberia. Owing to its excellent cold resistance and development potential, it has become an important introduced tree species in the Greater Xing’an area of China. Pine wilt disease, triggered by the pine wood nematode (PWN, *Bursaphelenchus xylophilus*), constitutes a profoundly critical affliction within forest ecosystems. Its incidence has extended to the northeastern region of China in recent years. To explore the potential host status of *P. sibirica* in the Greater Xing’an area for PWN and to elucidate the responses following inoculation, artificial inoculation, transcriptomics, and metabolomics methods were used. In the artificial inoculation experiments, quantitative analysis of nematode populations within the trees demonstrated that PWN exhibited normal growth and reproductive capabilities within *P. sibirica*. Subsequently, transcriptome and metabolome sequencing were conducted at four time points before disease onset (3-, 5-, 7-, and 9-days post inoculation). Gene trend analysis and differentially expressed gene screening were employed and the results indicated that genes associated with the flavonoid biosynthesis pathway exhibited predominant enrichment among the up-regulated genes. Metabolome analysis showed that the abundance of flavonoid-related metabolites in *P. sibirica* increased after inoculation with PWN. Integrated analysis of transcriptome and metabolome revealed that after PWN inoculation in *P. sibirica*, two *chalcone synthase* (*chs*) genes and a *chalcone isomerase* (*chi*) gene were significantly upregulated, and the upregulation should accumulate naringenin, pinocembrin, and apigenin to help *P. sibirica* resist infection of PWN. The results suggested that flavonoid biosynthesis pathway continued to respond after *P. sibirica* was infected with PWN and played an important role in the interaction between *P. sibirica* and PWN.

## 1 Introduction

*Pinus sibirica* is prominently distributed in the Siberian region, with its growth range spanning from 49°40′E to 127°20′E longitude and 46°30′N to 68°30’N latitude. Its distribution extends even to the forest limit within the Arctic Circle, demonstrating its strong ability to withstand cold (Hu and Luo, 2022). Since 1990, China has successively introduced *P. sibirica* to various regions, including Greater Xing’an Mountains, Lesser Xing’an Mountains, and Changbai Mountains. Many introduced *P. sibirica* forests have started to bloom and bear fruit (Wang et al., 2019).

The pine wood nematode (PWN, *Bursaphelenchus xylophilus*) is one of the major threats facing coniferous forests worldwide, causing a highly serious forest disease known as pine wilt disease (Futai, 2013). The nematode causes millions of dollars in losses annually and has been listed as a quarantine organism in many regions. Following its introduction to East Asia, the PWN triggered catastrophic consequences in pine forests, attributed to shifts in hosts, vector insects, and environmental conditions (Ye and Wu, 2022). PWN has the ability to survive in environments with annual average temperatures below 10°C. In recent years, PWN expanded its reach into Liaoning and Jilin in northeastern China (Zhao et al., 2017; Hou et al., 2021; Li et al., 2022). Notably, upon entering Liaoning, it was determined that *Pinus koraiensis* serves as a natural host for the nematode (Yu and Wu, 2018). However, there is currently no documented evidence suggesting that *P. sibirica* functions as a natural host for the PWN, it is necessary to conduct in-depth research on its resistance to PWNs given its importance.

Despite the numerous hypotheses proposed regarding the pathogenic mechanism of the PWN (Yang et al., 2003), the process of infection by the host plant remains unclear. Typically, plants develop an immune system as a defense mechanism against potential pathogens (Jones et al., 2016; Ngou et al., 2022). Plants respond to infection through two interrelated levels (Ngou et al., 2021; Yuan et al., 2021). At one level, plants recognize pathogen (microbe)-associated molecular patterns (PAMPs or MAMPs) through pattern recognition receptors (PRRs), a phenomenon known as PAMP (or MAMP)-triggered immunity (PTI or MTI) (Jones et al., 2016; Ngou et al., 2021; Yuan et al., 2021; Ngou et al., 2022). Subsequently, pathogens develop different virulence factors, referred to as effectors, to evade or inhibit PTI/MTI and successfully infect the host (Jones et al., 2016; Ngou et al., 2022). At another level, plants respond to microbe-specific effectors recognized by NB-LRR proteins, a process referred to as effector-triggered immunity (ETI) (Jones et al., 2016; Ngou et al., 2021; Yuan et al., 2021; Ngou et al., 2022). Within these two levels, plants initiate diverse downstream defense signal transduction pathways, encompassing cell surface immune receptors, intracellular immune receptors, mitogen-activated protein kinases, transcription factors, hormone signaling, etc., along with metabolic pathways, including secondary metabolism (Glazebrook, 2005; Kanyuka and Rudd, 2019).

Secondary metabolites are critically involved in plant defense mechanisms (Piasecka et al., 2015; Erb and Kliebenstein, 2020). Phenolic compounds, for instance, contribute to the establishment of a baseline resistance in plants (Treutter, 2005; Sudheeran et al., 2020). Certain flavonoids possess antibacterial, insecticidal, and antioxidant activities, while some ether compounds have demonstrated strong nematicidal activity (Dao et al., 2011; Hwang et al., 2021). Our previous research also indicated that chalcone synthase (*chs*) genes are vital in the resistance of *Pinus thunbergii* and *Pinus massoniana* against PWNs (Chen et al., 2021). Furthermore, it was found that the flavonoid compound catechin, possessing antioxidant capabilities, can reduce oxidative stress in *P. koraiensis*, thereby alleviating its symptoms (Zhang et al., 2022).

In this study, the artificial inoculation method was employed to analyze the symptoms of *P. sibirica* after inoculation with PWNs and the changes in nematode quantities within the trees. Transcriptome sequencing was employed for comparing differentially expressed genes after PWN infection, and identifying genes related to resistance against PWN infection. The metabolome analysis was utilized to determine changes in metabolites of *P. sibirica* after PWN infection. This research aims to explore the physiological response of *P. sibirica* to PWN infection, providing a reference for the investigation of pathogenicity and molecular mechanisms in the interactions between PWN and *P. sibirica*.

## 2 Materials and methods

### 2.1 Biological materials and PWN inoculation

Five-year-old *P. sibirica* seedlings used in the experiment were cultivated in the greenhouse of Northeast Forestry University, maintained at 23–28°C and a relative humidity of 65%–75%. PWNs (collected from Fushun City, Liaoning Province, China) were cultured in the dark at 25°C using *Botrytis cinerea*, which was cultured on potato dextrose agar medium. Nematodes (males: females: juveniles = 1:1:2) were isolated and collected using Baermann funnels to prepare a nematode suspension (3000 nematodes/100 μl). Following the method described in our previous research (Chen et al., 2021), PWNs were inoculated into *P. sibirica* seedlings (100 μl per plant) as the treatment group and isovolumes of ddH_2_O were inoculated into *P. sibirica* seedlings as the control check (CK) group.

All the treated and CK *P. sibirica* seedlings were divided into three groups. For the first group, five treated *P. sibirica* seedlings were randomly selected every day, and nematodes in the plants were collected using a Baermann funnel, then changes in the nematode population were calculated. For the second group, five treated and five CK *P. sibirica* seedlings were randomly selected every day, and stem segments were cut for plant tissue observation. For the third group, five treated and five CK *P. sibirica* seedlings were randomly selected every day, and 3 cm long stem segments were extracted from 1 cm below the inoculation site of each seedling, then all the segments were promptly placed in liquid nitrogen and maintained at −80°C for transcriptome sequencing and metabolome analysis.

### 2.2 RNA extraction, cDNA synthesis, library preparation, and sequencing

Total RNA extraction from both inoculated and control tree samples was conducted utilizing the RN38 EASYSpin Plus Plant RNA Kit (Aidlab Biotech, Beijing, China). Quantification and assessment of purity for total RNA were performed using the Bioanalyzer 2100 and RNA 1000 Nano LabChip Kit (Agilent, CA, USA), ensuring a RIN number > 7.0. Poly(A) RNA was isolated from total RNA (5 μg) through two rounds of purification using Poly-T oligomer magnetic beads. The purified mRNA underwent decomposition into smaller fragments under high-temperature conditions in the presence of divalent cations. Then, according to the mRNASeq sample preparation kit (Illumina, San Diego, CA, USA), the cleaved RNA fragments were reverse-transcribed to obtain the final cDNA library. The average insertion length of the paired library was 300 bp ( ± 50 bp). Sequencing was carried out using Illumina Novaseq™ 6000 (LC Sciences, Houston, TX, USA) following the recommended procedures for double c-terminal sequencing.

### 2.3 *De novo* assembly, unigene annotation, and functional classification

To ensure the acquisition of high-quality reads, the in-house Cutadapt (Kechin et al., 2017) and perl (https://registry.hub.docker.com/_/perl/) scripts were employed to eliminate adapter contamination, low-quality base, and reads with undetermined base. The quality of sequences was subsequently assessed using FastQC (http://www.bioinformatics.babraham.ac.uk/projects/fastqc/), encompassing Q20, Q30, and GC content of the clean data. All downstream analyses were exclusively conducted based on this high-quality clean data. *De novo* transcriptome assembly was carried out using Trinity 2.4.0 (Grabherr et al., 2011). In Trinity, transcripts were grouped into clusters based on shared sequence content, often loosely referred to as “genes”. The longest transcript within each cluster was designated as the representative “gene” sequence, also referred to as a single gene.

All assembled unigenes were associated with the non-redundant (Nr) protein database (http://www.ncbi.nlm.nih.gov/), the Gene Ontology (GO, http://www.geneontology.org), the SwissProt (http://www.expasy.ch/sprot/), the Kyoto Encyclopedia of Genes and Genomes (KEGG, http://www.genome.jp/kegg/) and the eggnog (http://eggnogdb.embl.de/) databases through DIAMOND (Buchfink et al., 2015), employing a threshold of *E* value < 0.00001.

### 2.4 Identification of differentially expressed genes

Expression levels for unigenes were quantified using Salmon (Patro et al., 2017) by calculating Transcripts Per Kilobase of exon model per Million mapped reads (TPM) (Mortazavi et al., 2008). The differentially expressed Unigenes were selected with log_2_ (fold change) > 1 or log_2_ (fold change) < -1 and with statistical significance (*P* value < 0.05) by R package edgeR (Robinson et al., 2010).

### 2.5 Mfuzz analysis

Advanced Mfuzz analysis was performed using the OmicStudio tools at https://www.omicstudio.cn/tool.

### 2.6 Metabolome analysis

After thawing the collected samples on ice, metabolites were isolated using an 80% methanol buffer. The Liquid Chromatograph Mass Spectrometer (LC-MS) system acquired all samples, following machine orders. Initially, all chromatographic separations were executed utilizing the UltiMate 3000 UPLC System (Thermo Scientific, Bremen, Germany). Reversed-phase separation occurred on an ACQUITY UPLC T3 column (100 mm × 2.1 mm, 1.8 μm, Waters, Milford, USA). The detection of metabolites eluted from the column was accomplished using the high-resolution tandem mass spectrometer Q-Exactive (Thermo Scientific, Bremen, Germany).

The MS data underwent various pretreatments, encompassing peak picking, peak grouping, retention time correction, second peak grouping, and annotation of isotopes and adducts utilizing XCMS (http://metlin.scripps.edu/download/) (Smith et al., 2006). LC-MS raw data files were transformed into mzXML format and processed through the XCMS, CAMERA (Kuhl et al., 2012), and metaX (http://metax.genomics.cn/) (Wen et al., 2017) toolbox integrated with the R software. The online KEGG and Human Metabolome Database (HMBD, https://hmdb.ca) were utilized for metabolite annotation by aligning the exact molecular mass data (m/z) of samples with those presented in the database. Additionally, an in-house fragment spectrum library of metabolites was employed to validate metabolite identification.

### 2.7 Identification of differentially abundant metabolites

Student *t*-tests were executed to identify variations in metabolite concentrations between 2 phenotypes. The *P* value was adjusted utilizing a false discovery rate (FDR) for multiple tests. Supervised partial least squares-discriminant analysis (PLS-DA) was executed utilizing metaX to discern distinct variables between groups. The variable important for the projection (VIP) value was computed. A VIP cut-off value of 1.0 was utilized for the selection of the significant features. Using the criteria of a fold change ≥ 1.5 or ≤ 1/1.5, *P* value ≤ 0.05, and VIP ≥ 1 to identify differentially abundant metabolites.

### 2.8 Real-time quantitative PCR analysis

RT-qPCR was performed with the GoTaq 2-Step RT-qPCR System Kit (Promega, Madison, WI, USA, catalogue number: A6010) and the Stratagene Mx3000P qPCR system (Agilent Technologies, Santa Clara, CA, USA) to validate the transcript levels of the genes (Chen et al., 2021). *Actin* was used as the internal control. All primers used in this study are listed in **Supplementary Table S1**. The normalization of the data was performed according to the instructions for the GoTaq 2-Step RT-qPCR System Kit and by the 2^−ΔΔC^_T_ method (Livak and Schmittgen, 2001). The experiments were repeated three times. Significance was determined by Student’s *t*-test.

## 3 Results

### 3.1 Changes in *P. sibirica* and nematode population after PWN inoculation

**Figure 1:**
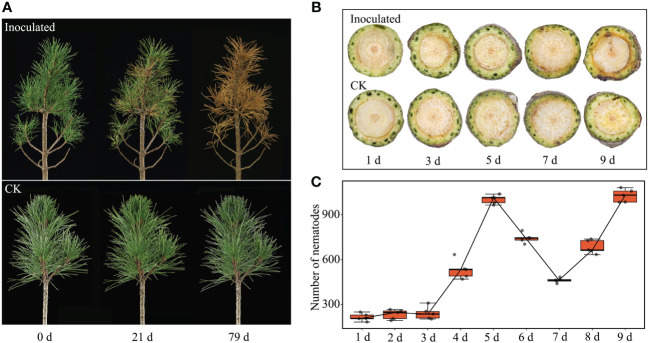
Changes of *P. sibirica* and PWN populations after inoculation. **(A)** Symptoms of *P. sibirica* after PWN inoculation. **(B)** Changes of *P. sibirica* segments 1 cm below inoculation sites. **(C)** Changes of PWN populations in *P. sibirica* after inoculation. CK, control check; indicated *P. sibirica* inoculated with an isovolume of ddH_2_O.

Symptoms in *P. sibirica* seedlings inoculated with PWNs became apparent at 21 days post inoculation (dpi), with approximately 25% of the needles displaying chlorosis. All inoculated *P. sibirica* seedlings showed symptoms by 27 dpi, with three-quarters of the needles exhibiting substantial yellowing. At 79 dpi, the inoculated *P. sibirica* seedlings had withered and died. The duration from the onset of symptoms to complete wilting and death spanned 58 days. Conversely, the CK of *P. sibirica* seedlings displayed no notable changes throughout the entire observation period (**Figure 1A**).

Observations from plant tissue sections of *P. sibirica* seedlings revealed that stem segments near the point of nematode inoculation initially became desiccated and shriveled. Symptoms of wilting appeared 1 cm below the inoculation site at 9 dpi (**Figure 1B**). At this stage, there were no apparent external changes in the *P. sibirica* seedlings (**Supplementary Figure S1**). Thus, internal symptoms in *P. sibirica* infected by PWNs appeared more quickly than external symptoms. Examination of the nematode population within the inoculated *P. sibirica* seedlings showed a decrease in nematode numbers at 5 dpi, possibly due to the mass mortality of adult nematodes following completion of their life cycle. Subsequently, nematode numbers gradually increased at 7 dpi, indicating the hatching of new larvae and signifying that PWNs could grow and reproduce normally within *P. sibirica* seedlings (**Figure 1C**).

### 3.2 Transcriptomic data analysis

Based on the onset of internal stem segment symptoms occurring 9 days after PWN inoculation in *P. sibirica* seedlings, an analysis was conducted to examine changes in gene expression from nematode inoculation to the onset of symptoms. Samples were selected corresponding to time points when the change in nematode population trend shifted (3 dpi, 5 dpi, 7 dpi, 9 dpi), along with control samples for each time point, leading to a total of eight samples. For each selected time point, five randomly collected pine seedlings were mixed in equal amounts and then subjected to transcriptome sequencing. An average of 5.8425 GB of valid data was obtained from each sample (**Supplementary Table S2**). Upon assembling all samples, 136,125 transcripts, and 71,938 genes were acquired. These sequences had average lengths of 558 bp and 516 bp, N50 values of 1,489 bp and 1,526 bp, and GC ratios of 43.97% and 44.77%, respectively. All genes were matched against six databases, including KEGG, eggNOG, Swiss-Prot, Pfam, Nr, and GO, utilizing BLASTX to obtain comprehensive genetic information (**Supplementary Table S3**). Functional annotations were acquired for 41,287 genes with respect to GO (57.39%), 27,097 genes for KEGG (37.67%), 37,611 genes for Pfam (52.28%), 34,353 genes for Swiss-Prot (47.75%), 45,181 genes for eggNOG (62.81%), and 41,914 genes for NR protein database (58.26%).

### 3.3 Gene expression trend analysis

**Figure 2:**
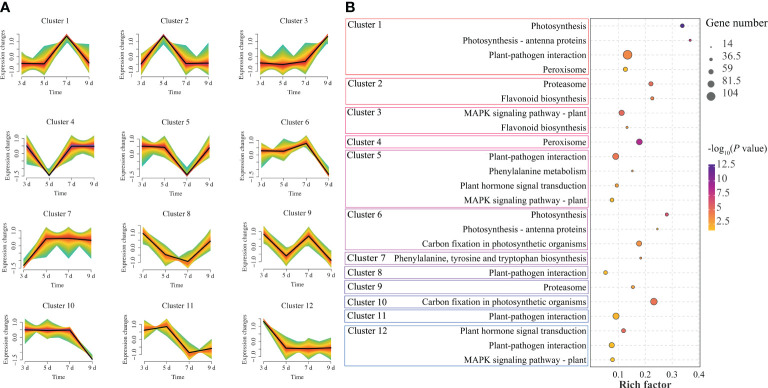
Gene expression trend analysis of *P. sibirica* after PWN inoculation. **(A)** Mfuzz expression trend analysis of gene expression changes. **(B)** KEGG enrichment results of highly enriched plant defense-related pathways in each cluster.

In order to explore the dynamic changes of genes in *P. sibirica* seedlings after PWN infection, the expression trends of all genes were analyzed and all genes were assigned to 12 clusters (**Figure 2A**; **Supplementary Table S4**). Among them, cluster 10 had the largest number of genes (8552 genes), and cluster 8 had the smallest number of genes (4611 genes). There was no obvious rule in the changes of gene expression patterns in different clusters. KEGG enrichment analysis was then performed on the genes in each cluster to further explore the differences in gene functions and genes significantly enriched in plant defense-related pathways were screened in each cluster (**Figure 2B**; **Supplementary Tables S5-16**). The results showed that all clusters had genes significantly enriched in plant defense-related pathways with high rich factor, indicating that genes related to plant defense-related pathways in *P. sibirica* seedlings might continue to respond after PWN infection.

### 3.4 Differentially expressed genes analysis

**Figure 3:**
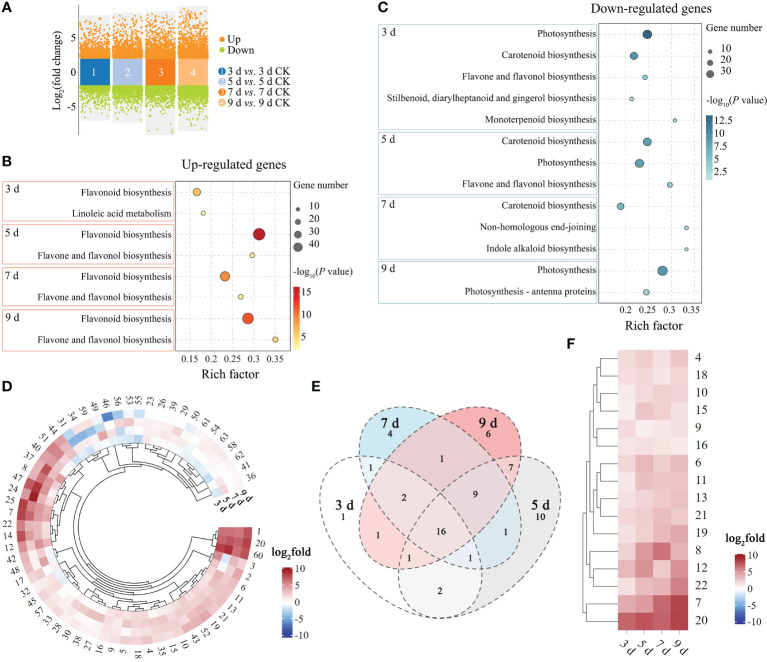
Differentially expressed genes analysis of *P. sibirica* after PWN inoculation. **(A)** Volcano plot of differentially expressed genes. **(B)** KEGG enrichment results of highly enriched plant defense-related pathways by up-regulated genes. **(C)** KEGG enrichment results of highly enriched plant defense-related pathways by down-regulated genes. **(D)** Expression change heatmap of up-regulated genes enriched in the flavonoid biosynthesis pathway. **(E)** Venn diagram of up-regulated genes enriched in the flavonoid biosynthesis pathway. **(F)** Expression change heatmap of genes enriched in the flavonoid biosynthesis pathway that were up-regulated at all time points.

Gene expression trend analysis showed that genes related to plant defense-related pathways in *P. sibirica* seedlings exhibited different changing trends after PWN infection. To determine whether the differential expression changes of these genes were significant and to screen for genes associated with the PWN inoculation in *P. sibirica* seedlings, genes differentially expressed after inoculation were analyzed. The results showed that there were 4679 genes exhibiting differential expression at 3 dpi, with 2531 genes up-regulated and 2148 genes down-regulated. At 5 dpi, 6962 genes showed differential expression, with 3280 genes up-regulated and 3682 genes down-regulated. At 7 dpi, 6098 genes exhibited differential expression, including 3068 genes up-regulated and 3030 genes down-regulated. At 9 dpi, 8100 genes showed differential expression, with 3981 genes up-regulated and 4119 genes down-regulated (**Figure 3A**; **Supplementary Table S17**).

KEGG enrichment analysis was then performed on differentially expressed genes at different time points. Each time point had differentially expressed genes enriched in multiple plant defense-related pathways (**Supplementary Tables S18-25**). However, it cannot be ignored that each time point had a large number of up-regulated genes enriched in flavonoid biosynthesis (ko00941) with high rich factor (**Figure 3B**), indicating that flavonoid biosynthesis continued to respond in *P. sibirica* seedlings after PWN inoculation. And each time point had a large number of down-regulated genes enriched in photosynthesis (ko00195), photosynthesis-antenna proteins (ko00196) and carotenoid biosynthesis (ko00906) with high rich factors (**Figure 3C**), indicating that photosynthesis continued to be inhibited in *P. sibirica* seedlings after PWN inoculation.

### 3.5 Plant defense-related genes selection

The KEGG enrichment results of differentially expressed genes indicated that the flavonoid biosynthesis pathway might respond quickly and sustainably in *P. sibirica* seedlings after PWN inoculation. Therefore, the expression patterns of up-regulated genes that enriched in the flavonoid biosynthesis pathway at each time point were further analyzed to explore whether these genes were associated with the inoculation time. After removing duplicate genes, a total of 63 genes enriched in the flavonoid biosynthesis pathway were found to be up-regulated at any time point (**Supplementary Table S26**). Expression pattern analysis showed that most of these genes were up-regulated at multiple time points, and only a few were down-regulated at some time points (**Figure 3D**). Among them, 16 genes were significantly up-regulated at all time points (**Figures 3E, F**; **Supplementary Table S27**). Ten genes were randomly selected from these 16 genes for RT-qPCR verification, including three *chalcone-related* genes (No. 11, 19 and 20), two *caffeoyl-CoA O-methyltransferase* genes (No. 6 and 10), a *pinosylvin synthase* gene (No. 7), an *anthocyanidin reductase* gene (No. 13), a *flavonol synthase* gene (No. 16), a *shikimate O-hydroxycinnamoyltransferase* gene (No. 4), and a *bifunctional dihydroflavonol 4-reductase/flavanone 4-reductase* gene (No. 21). The trends of RT-qPCR results and RNA-seq results of the 10 genes were consistent (**Supplementary Figure S2**), indicating the reliability of the transcriptome sequencing and the importance of these genes in *P. sibirica* seedlings after PWN inoculation.

### 3.6 Metabolome analysis

**Figure 4:**
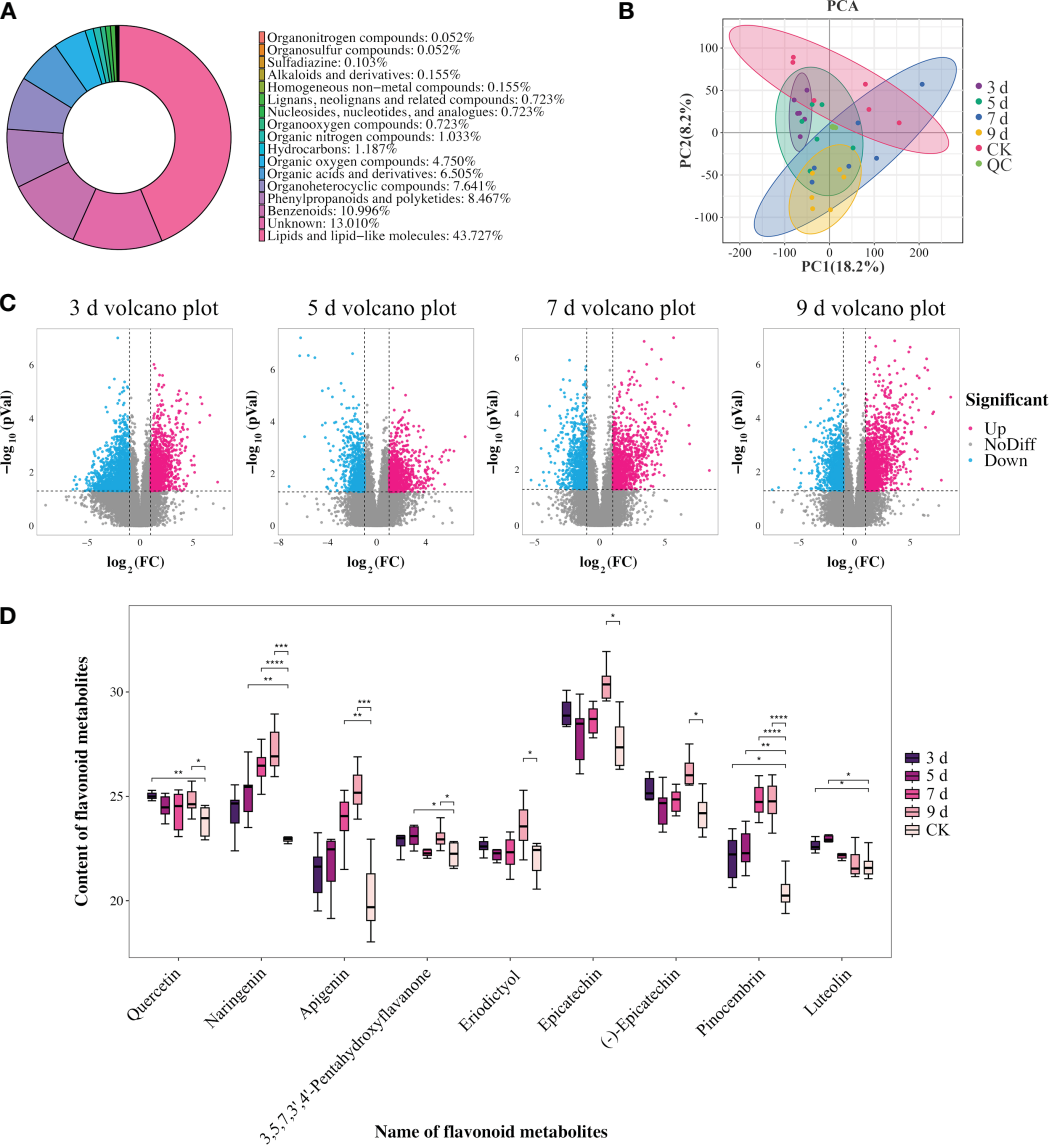
Identification and analysis of differentially abundant metabolites in *P. sibirica* after PWN inoculation. **(A)** Categories of identified metabolites in non-targeted metabolomic analysis. **(B)** Principal component analysis of identified metabolites. **(C)** Statistical analysis of the number of differentially abundant metabolites. **(D)** Content changes of nine selected flavonoid-related metabolites. Analyzed using one-way ANOVA and *t*-test, with asterisks denoting significant differences. One asterisk represents *P*<0.05, two asterisks represent *P*<0.01, three asterisks represent *P* < 0.001, and four asterisks represent *P* < 0.0001.

In order to further explore the role of flavonoid biosynthesis in *P. sibirica* seedlings after PWN inoculation, non-targeted metabolomic analysis was executed to identify the differentially abundant metabolites at 3, 5, 7, and 9 dpi. A total of 1937 metabolites were identified and classified into 15 categories, with lipids and lipid molecules being the predominant metabolites (**Figure 4A**; **Supplementary Table S28**). For a comprehensive understanding of the metabolic changes during the infection by PWNs at the four different time points, a quantitative analysis was performed on the metabolome data. Principal component analysis showed that, except for at 9 dpi, the other samples could not be effectively distinguished from the control sample (**Figure 4B**). At 9 dpi, the samples intersected with those at 7 dpi and 5 dpi while remaining distinct from 3 dpi (**Figure 4B**). This indicated that only the metabolome data at 9 dpi had a high degree of differentiation from the control. Furthermore, the metabolome data at 9 dpi exhibited a higher degree of differentiation from the metabolome data at 3 dpi. These results indicated that the metabolic impact on *P. sibirica* seedlings was relatively minor at 3 dpi. In contrast, the impact of PWNs on the metabolism of *P. sibirica* seedlings was notably significant during the early stages of disease at 9 dpi.

### 3.7 Differentially abundant metabolite analysis

Subsequently, using the criteria of the methods, differentially abundant metabolites were identified when comparing 3, 5, 7, and 9 dpi with the control. A comprehensive analysis of these differentially abundant metabolites was conducted across the different comparisons (**Figure 4C**; **Supplementary Table S29**). The statistical examination revealed 347 significantly differentially abundant metabolites between 3 dpi and the control, encompassing 138 upregulated and 209 downregulated metabolites. When comparing metabolites from 5 dpi sample with the control, 210 significantly differentially abundant metabolites were observed, including 116 increased and 94 decreased metabolites. Approximately 237 significantly differentially abundant metabolites were observed between metabolites from 7 dpi sample and the control, with 142 increased and 95 decreased metabolites. Finally, the comparison of metabolites from 9 dpi sample with the control unveiled 266 differentially abundant metabolites, comprising 168 increased and 98 decreased metabolites.

Following this, KEGG enrichment analysis was executed on the differentially abundant metabolites within these comparative groups. Subsequently, all identified differentially abundant metabolites were assigned to distinct metabolic pathways (**Supplementary Figure S3**). At 3 dpi, the pathways implicated included glycerolipid metabolism (ko00561), pantothenate and CoA biosynthesis (ko00770), glycosylphosphatidylinositol (GPI)-anchor biosynthesis (ko00563), plant hormone signal transduction (ko04075), and flavone and flavonol biosynthesis (ko00944) (**Supplementary Figure S3**, **Supplementary Table S30**). At 5 dpi, the enriched pathways for differentially abundant metabolites included glycosylphosphatidylinositol (GPI)-anchor biosynthesis (ko00563), flavonoid biosynthesis, biosynthesis of phenylpropanoids (ko01061), alpha-linolenic acid metabolism (ko00592), and galactose metabolism (ko00052) (**Supplementary Figure S3**, **Supplementary Table S31**). At 7 dpi, the observed differentially abundant metabolites were allocated to ABC transporters (ko02010), phenylpropanoid biosynthesis (ko00940), alpha-linolenic acid metabolism (ko00592), arginine biosynthesis (ko00220), and biosynthesis of phenylpropanoids (**Supplementary Figure S3**, **Supplementary Table S32**). Finally, at 9 dpi, the differentially abundant metabolites were linked to flavonoid biosynthesis, ABC transporters, arginine biosynthesis, biosynthesis of phenylpropanoids, and alanine, aspartate and glutamate metabolism (ko00250) (**Supplementary Figure S3**, **Supplementary Table S33**).

### 3.8 Integrated analysis of transcriptome and metabolome

The transcriptome analysis results showed that differentially expressed genes significantly enriched in flavonoid biosynthesis, flavone and flavonol biosynthesis, and phenylpropanoid biosynthesis pathways, suggesting that flavonoid-related pathways might play an important role in *P. sibirica* seedlings after PWN inoculation. Similarly, as differentially abundant metabolites were significantly enriched in flavonoid biosynthesis at 5 dpi and 9 dpi samples, in flavone and flavonol biosynthesis at 3 dpi, and in phenylpropanoid biosynthesis at 7 dpi, differentially abundant metabolites enriched in flavonoid-related pathways were selected at various time points for further research (**Figure 4D**). Upon screening, it became evident that all differentially abundant flavonoid-related metabolites were increased. Specifically, the increased flavonoid metabolites reached the highest count of 9 at 9 dpi, followed by 5 dpi and 3 dpi with counts of 5, and the lowest count of 3 at 7 dpi. Notably, pinocembrin was increased at 3, 5, 7, and 9 dpi, while naringenin was increased at 5, 7, and 9 dpi, and apigenin was increased at 7 and 9 dpi. In addition, the levels of these three metabolites steadily increased each day after inoculation. It was suggested that the sustained significant increases of these metabolites in the late stages of PWN inoculation should have contributed to the resistance of *P. sibirica* seedlings to PWN infection.

**Figure 5:**
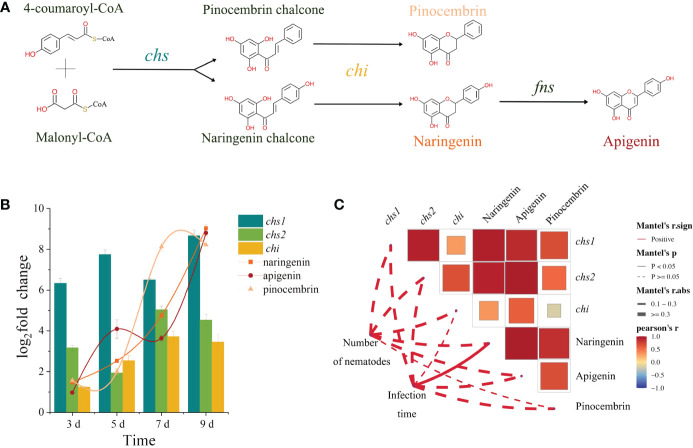
Integrated analysis of transcriptome and metabolome. **(A)** The synthesis pathway of pinocembrin, naringenin, and apigenin. **(B)** Changes in the expression levels of *chs1*, *chs2* and *chi*, and the abundance levels of pinocembrin, naringenin, and apigenin. **(C)** Correlation analysis of expression levels of *chs1*, *chs2* and *chi*, and abundance levels of pinocembrin, naringenin and apigenin with inoculation time and the number of nematodes in the *P. sibirica* seedlings after PWN inoculation. The red line represents the positive correlation, the line form represents the significance value, and the thickness of the line represents the correlation value. The color of the box represents the correlation value, and the size of the box represents the significance value.

By analyzing the synthesis pathways of these three metabolites, it became apparent that naringenin and pinocembrin shared consistent prerequisites and enzymes required for their synthesis, suggesting a competitive relationship between them. In contrast, apigenin was identified as a downstream metabolite of naringenin (**Figure 5A**). Through RT-qPCR and metabolome analysis, the three genes [two *chalcone synthase* (*chs*) genes (No. 19 and 20) and a *chalcone isomerase* (*chi*) gene (No.11)] and three metabolites (naringenin, pinocembrin and apigenin) screened out in the pathway map were analyzed. Analysis of expression levels showed that the expression of *chs1*, *chs2* and *chi* continued to increase in *P. sibirica* seedlings after PWN inoculation, and with the up-regulation of the three genes, the contents of naringenin, pinocembrin and apigenin also increased significantly (**Figure 5B**). Next, we conducted a correlation analysis between expression levels of *chs1*, *chs2* and *chi*, and abundance of naringenin, pinocembrin and apigenin, with the inoculation time and the number of nematodes in the *P. sibirica* seedlings after PWN inoculation. The results showed that the three genes and the three metabolites were positively correlated with the inoculation time and the number of nematodes in the seedlings (**Figure 5C**).

## 4 Discussion

In the 1950s, forestry professionals discovered scattered pines on the northern slope of the Greater Xing’an Mountains, mistakenly identifying them as *P. koraiensis* (Liu et al., 2002). However, subsequent research refuted this identification, demonstrating that *P. koraiensis* cannot survive in the Greater Xing’an Mountains. They were not *P. koraiensis* but rather *P. sibirica* (Wang et al., 2019). *P. sibirica* is a highly valuable tree species, with its wood and fruits having high utility value (Liu et al., 2002; Wang et al., 2019). Its light, soft, and fine-grained wood makes it easy to process and is commonly used in construction and furniture manufacturing industries. In addition, its pine nuts contain rich nutrients, offering many health benefits. Therefore, the development potential of *P. sibirica* is enormous (Ji et al., 2023). However, with the continuous northward expansion of PWNs, *P. sibirica* faces significant survival challenges. Furthermore, *P. sibirica* has a close relationship with *P. koraiensis* (Wang et al., 2019), implying that it might serve as a natural host for PWN.

This study found that under artificial inoculation conditions, *P. sibirica* begins to show symptoms at 9 dpi and eventually died at 79 dpi. Examination of nematode counts in the early stages of infection reveals that PWN can grow and reproduce normally in *P. sibirica*. Therefore, investigating the response of *P. sibirica* to the infection of PWN is extremely important. Transcriptomics proves invaluable in identifying differentially expressed genes that reflect the host gene response in the interaction between host and pathogen. Hence, this study utilized transcriptomic techniques to analyze the differentially expressed gene expression in *P. sibirica* at 3, 5, 7, and 9 dpi with PWN.

Numerous studies provide evidence that pine trees undergo genetic changes over time in response to PWN infection. For instance, in *P. massoniana*, through transcriptomic data analysis, the core gene module in the early stage of response to PWN was identified, and genes closely related to this module were found to play important roles in oxidative phosphorylation, amino sugar, and nucleotide sugar metabolism, as well as the plant MAPK signaling pathway (An et al., 2023). In this study, after expression trend analysis, all genes were divided into 12 clusters. KEGG enrichment analysis was performed on the genes in these clusters, and the results showed that each cluster had genes enriched in in plant defense-related pathways, including plant-pathogen interaction, MAPK signaling pathway–plant, and flavonoid biosynthesis. The results were consistent with previous studies. Some researchers observed a significant decrease in net light and rate, chlorophyll content, and stomatal conductance following PWN infection in pine trees, indicating that PWN may cause damage to the photosynthetic structure of pine needles (Liu et al., 2023). In this study, a large number of down-regulated genes were enriched in photosynthesis (ko00195), photosynthesis-antenna proteins (ko00196) and carotenoid biosynthesis (ko00906) with high rich factors (**Figure 3C**), indicating that photosynthesis continued to be inhibited in *P. sibirica* seedlings after PWN inoculation.

Pathways involved in carbohydrate metabolism were also found to be highly enriched in, such as glycolysis/gluconeogenesis and starch and sucrose metabolism. This matched previous studies, which found that when PWN invaded pine trees, changes occurred in carbohydrate metabolism within the tree, including decreased sucrose levels, resulting in the accumulation of glucose and fructose at the infected site. The accumulation of these metabolites might further be related to the induced expression of pathogenesis-related genes (Proels and Hückelhoven, 2014; Rojas et al., 2014). This study also found that plant defense-related pathways might continue to respond in *P. sibirica* after PWN inoculation. Prior studies had demonstrated that primary metabolism is critically involved in meeting the energy demands of plant defense mechanisms and serves as a source of signaling molecules in response to biotic stress (Berger et al., 2007; Bolton, 2009; Steinbrenner et al., 2011; Rojas et al., 2014). Similarly, secondary metabolites act as essential regulators in plant-environmental interactions and biological stress defense responses (Piasecka et al., 2015; Erb and Kliebenstein, 2020). The results from this study combined with these findings suggest that metabolic changes are the primary response to PWN infection.

The results of KEGG enrichment analysis of differentially expressed genes at different time points also showed that genes enriched in the flavonoid biosynthesis pathway had the characteristics of rapid response and long sustained reaction time. Similarly, our previous research also indicated that the expression of most genes, encompassing plant defense-related genes, including those associated with plant hormone signal transduction, plant-pathogen interactions, and the MAPK signaling pathway in *P. thunbergii* and *P. massoniana*, was downregulated after PWN infection (Chen et al., 2021). Conversely, numerous chalcone synthase genes and their associated genes showed sustained elevation in expression post-PWN infection (Chen et al., 2021). Combined with previous research results, we believe that the flavonoid biosynthesis-related pathways may continue to respond in *P. sibirica* after PWN inoculation, and a metabolome analysis was used to test this hypothesis.

The results from untargeted metabolomics showed that differentially abundant metabolites of *P. sibirica* after PWN inoculation enriched in the flavonoid biosynthesis-related pathways at various time points. Analysis of the levels of the metabolites enriched in the flavonoid-related pathways revealed that all differentially abundant flavonoid metabolites were upregulated. Specifically, for apigenin, naringenin, and pinocembrin, which showed consistently increased expression levels, the examination in conjunction with transcriptomic studies indicated that the upregulation of *chs* genes in the transcriptome might lead to the accumulation of pinocembrin chalcone and naringenin chalcone. Additionally, the upregulated *chi* gene, using the accumulated pinocembrin chalcone and naringenin chalcone as substrates, might promote the accumulation of pinocembrin and naringenin, thereby increasing their content. Moreover, due to the accumulation of naringenin, apigenin might rapidly accumulate, thereby increasing its own content. The correlation between the three genes and the three metabolites were then analyzed with the inoculation time and the number of nematodes in the *P. sibirica* seedlings after PWN inoculation. The positive correlation between them indicated that the changes in the expression levels of *chs-1*, *chs-2* and *chi* were consistent with the upstream and downstream relationships between the changes in the abundance of naringenin, pinocembrin and apigenin, and that these changes were related to the inoculation of PWN.

The decline in tree vigor resulting from pine tree infection by PWN may make trees susceptible to mixed infections by other pathogens. Previous studies have shown that pinocembrin, naringenin, and apigenin exhibit antifungal activity, thereby inducing plant disease resistance (Danelutte et al., 2003; Elbatreek et al., 2023; Jurčević Šangut et al., 2023). Isolation of pinocembrin from resistant *Pinus strobus* against PWN has been reported, suggesting its role in conferring resistance (Hanawa et al., 2001). In addition to their antimicrobial activity, pinocembrin and naringenin also exhibit strong antioxidant capabilities (Hernández-Aquino and Muriel, 2018; Elbatreek et al., 2023). During the process of PWN infection in pine trees, a hypersensitive response is triggered. This response starts with an oxidative burst of reactive oxygen species (ROS), which is activated to restrict the growth of obligate parasitic pathogens (Averyanov, 2009; Nanda et al., 2010). While elevated levels of ROS production can be detrimental to cell integrity, they are also essential for plant defense (Heath, 2000; Nanda et al., 2010). Therefore, inhibiting ROS toxicity and controlling ROS buildup in plants are crucial for disease resistance (Mittler et al., 2004). Furthermore, catechin, which also possesses antioxidative capabilities, can delay disease onset in *P. koraiensis* (Zhang et al., 2022). It has also been revealed that pinocembrin can reduce lipid accumulation (Oikawa et al., 2016). Significantly, the lipid content of third-stage dispersal juvenile (DJ3), which is associated with low-temperature survival and high dispersal of PWN, is notably higher than that of other larval stages. Therefore, following PWN infection, pinocembrin in *P. sibirica* may prevent overwintering or dispersion of the nematodes by inhibiting the accumulation of DJ3’s lipids.

In summary, this research examines that PWNs can infect *P. sibirica* through artificial inoculation, demonstrating their capability for normal growth and reproduction within *P. sibirica*. A comparison of the internal onset time with the external symptomatic appearance time revealed that symptoms manifest internally in *P. sibirica* faster than their external symptomatic appearance. Combined with previous research results, we believed that the flavonoid biosynthesis pathway continued to respond after *P. sibirica* was infected with PWN and played an important role in the interaction between *P. sibirica* and PWN. Analysis of both transcriptome and metabolome results revealed that after PWN infection in *P. sibirica*, the *chs1*, *chs2*, and *chi* genes were significantly upregulated, and the upregulation should accumulate naringenin, pinocembrin, and apigenin to help *P. sibirica* resist infection of PWN.

## Data availability statement

The datasets presented in this study can be found in online repositories. The names of the repository/repositories and accession number(s) can be found in the article/**Supplementary Material**.

## Ethics statement

The manuscript presents research on animals that do not require ethical approval for their study.

## Author contributions

JZ: Conceptualization, Formal analysis, Investigation, Methodology, Validation, Visualization, Writing – original draft. LY: Investigation, Writing – original draft. QC: Conceptualization, Data curation, Formal analysis, Funding acquisition, Investigation, Methodology, Resources, Validation, Visualization, Writing – original draft, Writing – review & editing. FW: Funding acquisition, Project administration, Resources, Supervision, Writing – review & editing.
