# Supplementary material for: Response analysis of Pinus sibirica to pine wood nematode infection through transcriptomics and metabolomics study
Source: Front Plant Sci. 2024 May 7;15:1383018. doi: 10.3389/fpls.2024.1383018 (PMC11106439; doi:10.3389/fpls.2024.1383018)
Supplement: Supplementary file 1 [file DataSheet_1.pdf]

Supplementary Material

1 Supplementary Figures and Tables

1.1 Supplementary Figures

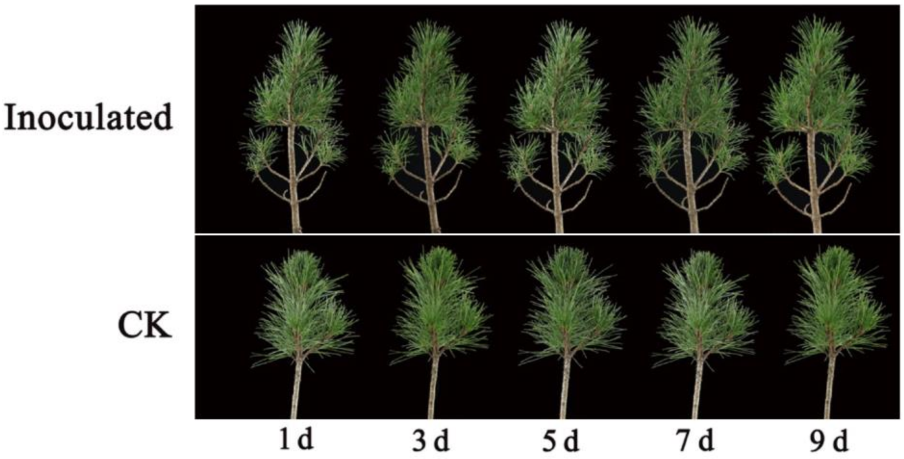

Supplementary Figure 1 Changes of *P. sibirica* at 1、3、5、7 and 9 days post inoculation.

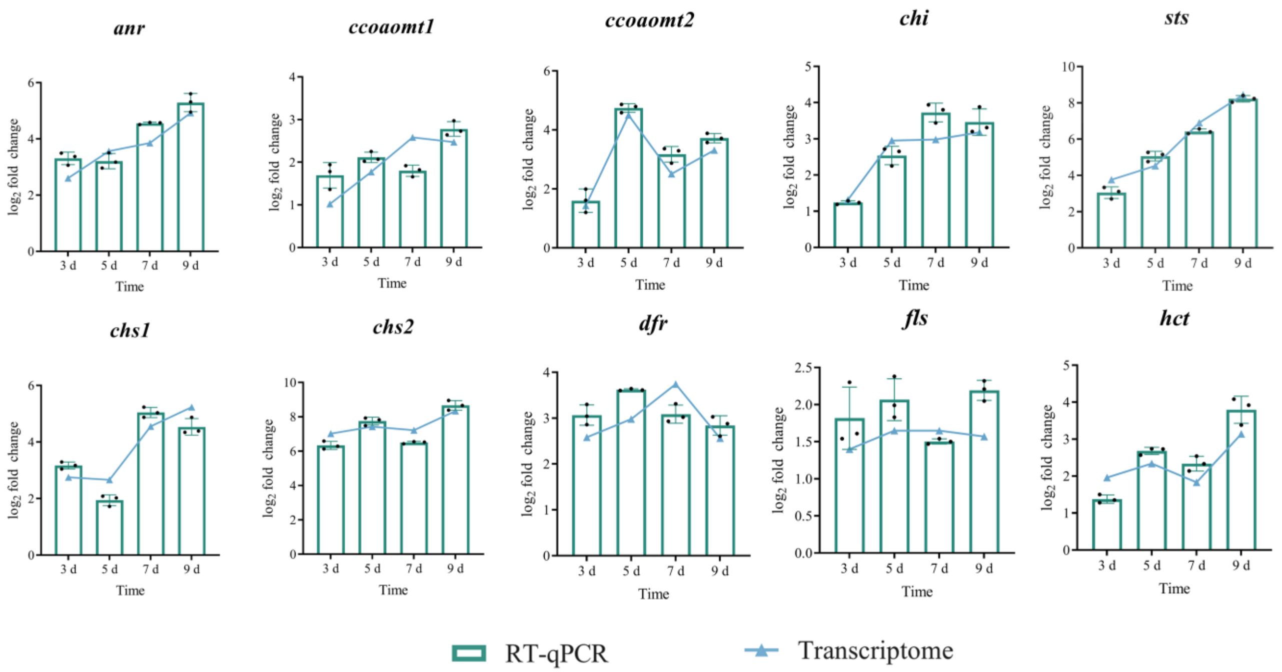

Supplementary Figure 2 Expression patterns of 10 flavonoid pathway-related genes.

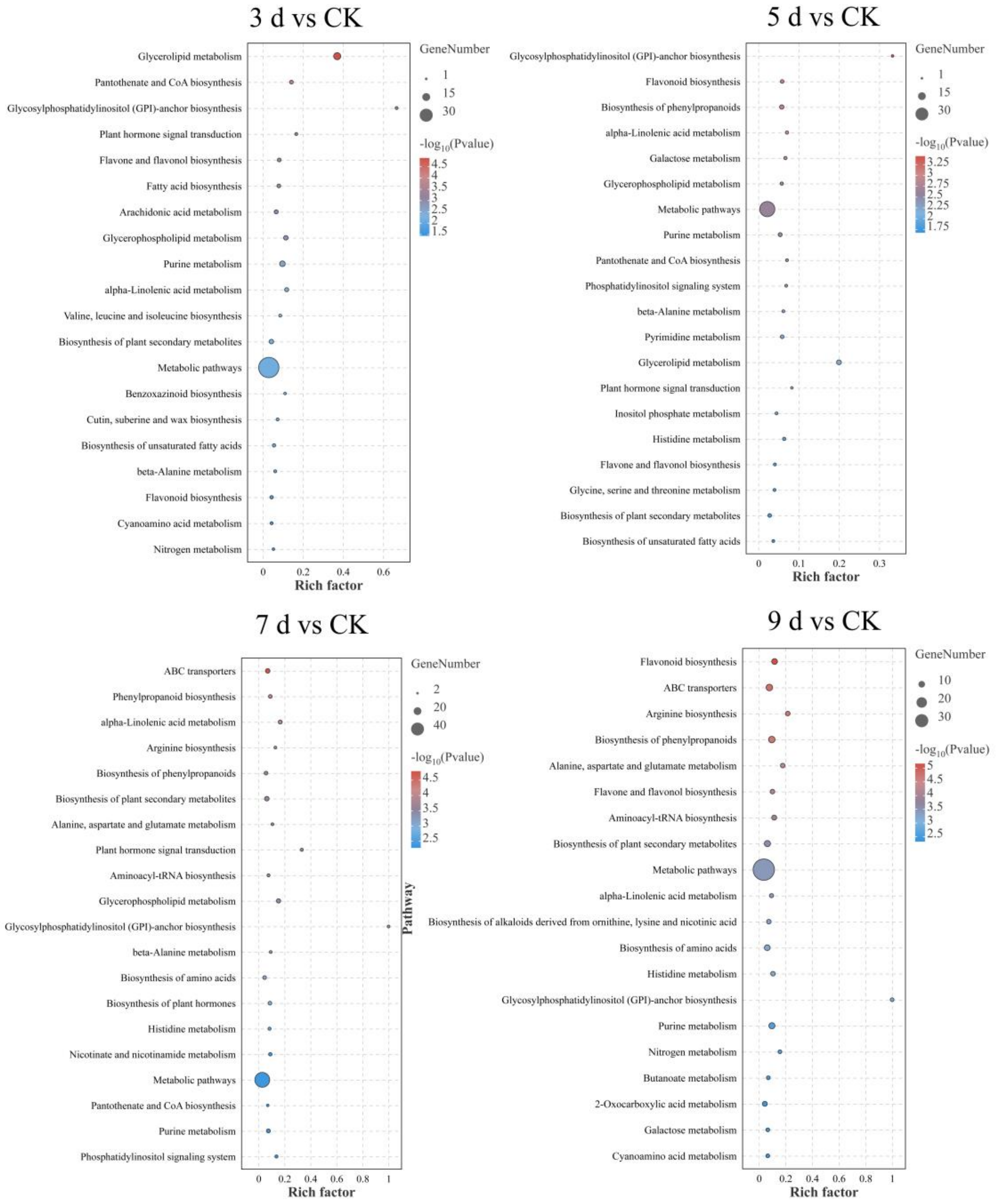

Supplementary Figure 3 Top 20 pathways with the smallest *P* values in KEGG enrichment outcomes in differentially accumulated metabolites.

1.2 Supplementary Tables

Supplementary Table 1 RT-qPCR primers used in this study.

Gene	Forward primer	Reverse primer
ccoaomt1	GTTCAAACAGGCAGCCACAG	GTCAGTTCCCGAAGCTCCTT
anr	GGACAAAGGCTACACTGTGC	TTGATGGCAGCGTCGAAAC
sts	GAAACTCCAGAGGGCAGATG	TGCCGACCTTTCACATATTCG
hct	ACCAGACCAGACCACAACAA	CCGATCTAAGCGCAACGAAA
ccoaomt2	CCCGCATTCATTGAGTCACA	TGCAAGAGACTTTTGTGGCC
fls	CCAGGAGTTGCAGGAGAGTT	TCGGAGTGTTTGGCATTTGG
dfr	AAGAGGGCGCATTTGATTCC	CACGCCTCACTGATTTAGCC
chs1	GAGGAGCTAGGGTTCTGGTC	CTCTCTCAGCTTCCCACCAA
chs2	GGAAAAGAAGCAGCCACCAA	TGACTCTCTTAACGCTGGGG
chi	ATAATTCGAGCACTGCCTGG	CGCCGTTGGAGCAATTGATT
Actin	GGCTTCATCCAATCCTCGAT	TGCGTCGATAACTACTGCCT

Supplementary Table 2 Quality statistics of filtered reads.

Sample	Total Raw Reads (b)	Total Clean Reads (b)	Total Clean Bases(Gb)	Clean Reads Q20(%)	Clean Reads Q30(%)	Clean Reads Ratio(%)
3 dpi	42874926	41912632	5.82	96.59	90.56	97.76
3 dpi CK	41392560	40548054	5.64	96.81	91.03	97.96
5 dpi	44987132	44118702	6.13	96.68	90.67	98.07
5 dpi CK	43552422	42684776	5.93	96.70	90.75	98.01
7 dpi	40198424	39206838	5.43	96.12	89.49	97.53
7 dpi CK	42587696	41596062	5.76	96.26	89.79	97.67
9 dpi	44448860	43384108	6.01	96.22	89.69	97.60
9 dpi CK	44402712	43405172	6.02	96.27	89.79	97.75

Supplementary Table 3 Gene annotation statistics table.

DB	Num	Ratio(%)
All	71938	100.00
GO	41287	57.39
KEGG	27097	37.67
Pfam	37611	52.28
swissprot	34353	47.75
eggNOG	45181	62.81
NR	41914	58.26

Supplementary Table 4 The number of genes in each cluster.

Cluster	Gene count
1	5918
2	6704
3	5223
4	8034
5	5603
6	5873
7	5037
8	4611
9	5794
10	8552
11	4961
12	5390

Supplementary Table 5 KEGG enrichment result for genes in cluster 1 (*P*<0.05).

KEGG A class	KEGG B class	Pathway	Count	Rich factor	*P* value	Pathway ID
**Metabolism**	**Energy metabolism**	**Photosynthesis**	**41**	**0.33884298**	**1.11e-13**	**ko00195**
**Metabolism**	**Energy metabolism**	**Photosynthesis - antenna proteins**	**21**	**0.36842105**	**2.06e-08**	**ko00196**
**Organismal Systems**	**Environmental adaptation**	**Plant-pathogen interaction**	**104**	**0.13471503**	**0.00012479**	**ko04626**
Metabolism	Carbohydrate metabolism	Glycolysis / Gluconeogenesis	84	0.13043478	0.00146244	ko00010
Genetic Information Processing	Folding, sorting and degradation	Protein processing in endoplasmic reticulum	109	0.11925602	0.00631481	ko04141
Metabolism	Global and overview maps	Biosynthesis of secondary metabolites	409	0.10425695	0.00956822	ko01110
Metabolism	Metabolism of other amino acids	Glutathione metabolism	38	0.13571429	0.01489132	ko00480
Organismal Systems	Environmental adaptation	Circadian rhythm - plant	17	0.16346154	0.0178254	ko04712
Metabolism	Lipid metabolism	Biosynthesis of unsaturated fatty acids	25	0.14619883	0.01881555	ko01040
Metabolism	Lipid metabolism	Fatty acid degradation	39	0.13265306	0.01936524	ko00071
Metabolism	Amino acid metabolism	Histidine metabolism	18	0.15789474	0.02095268	ko00340
**Cellular Processes**	**Transport and catabolism**	**Peroxisome**	**47**	**0.12737127**	**0.02187055**	**ko04146**
Metabolism	Carbohydrate metabolism	Pyruvate metabolism	65	0.11970534	0.02813986	ko00620
Metabolism	Global and overview maps	Metabolic pathways	697	0.09931604	0.03563353	ko01100
Metabolism	Global and overview maps	Biosynthesis of amino acids	94	0.11257485	0.04175164	ko01230
Metabolism	Metabolism of cofactors and vitamins	Riboflavin metabolism	7	0.2	0.04285163	ko00740
Metabolism	Amino acid metabolism	Arginine and proline metabolism	34	0.12781955	0.04371756	ko00330

Supplementary Table 6 KEGG enrichment result for genes in cluster 2 (*P*<0.05).

KEGG A class	KEGG B class	Pathway	Count	Rich factor	*P* value	Pathway ID
Genetic Information Processing	Replication and repair	DNA replication	38	0.35514019	7.10e-11	ko03030
Genetic Information Processing	Replication and repair	Homologous recombination	41	0.33064516	1.66e-10	ko03440
Genetic Information Processing	Replication and repair	Mismatch repair	25	0.30120482	4.14e-06	ko03430
**Genetic Information Processing**	**Folding, sorting and degradation**	**Proteasome**	**46**	**0.22222222**	**8.23e-06**	**ko03050**
Metabolism	Nucleotide metabolism	Pyrimidine metabolism	64	0.19047619	3.43e-05	ko00240
Genetic Information Processing	Replication and repair	Nucleotide excision repair	32	0.23703704	5.07e-05	ko03420
**Metabolism**	**Biosynthesis of other secondary metabolites**	**Flavonoid biosynthesis**	**34**	**0.22666667**	**7.86e-05**	**ko00941**
Genetic Information Processing	Replication and repair	Base excision repair	20	0.27027027	0.00019692	ko03410
Genetic Information Processing	Translation	Ribosome biogenesis in eukaryotes	51	0.18021201	0.00079527	ko03008
Metabolism	Glycan biosynthesis and metabolism	N-Glycan biosynthesis	24	0.20512821	0.00349233	ko00510
Genetic Information Processing	Transcription	Basal transcription factors	18	0.20930233	0.00853353	ko03022
Genetic Information Processing	Folding, sorting and degradation	SNARE interactions in vesicular transport	11	0.22916667	0.01875687	ko04130
Genetic Information Processing	Replication and repair	Non-homologous end-joining	5	0.33333333	0.02268372	ko03450
Metabolism	Metabolism of terpenoids and polyketides	Zeatin biosynthesis	7	0.26923077	0.02459045	ko00908
Metabolism	Glycan biosynthesis and metabolism	Glycosaminoglycan degradation	11	0.21153846	0.03276297	ko00531
Metabolism	Biosynthesis of other secondary metabolites	Stilbenoid, diarylheptanoid and gingerol biosynthesis	7	0.25	0.03612467	ko00945
Genetic Information Processing	Translation	Aminoacyl-tRNA biosynthesis	26	0.16352201	0.04217916	ko00970
Genetic Information Processing	Folding, sorting and degradation	Ubiquitin mediated proteolysis	54	0.14555256	0.04286055	ko04120
Genetic Information Processing	Folding, sorting and degradation	RNA degradation	46	0.1474359	0.04834021	ko03018

Supplementary Table 7 KEGG enrichment result for genes in cluster 3 (P<0.05 and top 20 pathways).

KEGG A class	KEGG B class	Pathway	Count	Rich factor	*P* value	Pathway ID
Metabolism	Global and overview maps	Metabolic pathways	542	0.07722998	6.82e-14	ko01100
Metabolism	Global and overview maps	Biosynthesis of secondary metabolites	309	0.07876625	2.70e-07	ko01110
Metabolism	Lipid metabolism	alpha-Linolenic acid metabolism	28	0.16374269	1.96e-06	ko00592
**Environmental Information Processing**	**Signal transduction**	**MAPK signaling pathway - plant**	**60**	**0.11257036**	**4.32e-06**	**ko04016**
Metabolism	Amino acid metabolism	Tryptophan metabolism	36	0.1220339	7.03e-05	ko00380
Metabolism	Lipid metabolism	Fatty acid degradation	35	0.11904762	0.00014464	ko00071
Metabolism	Amino acid metabolism	Valine, leucine and isoleucine degradation	31	0.11742424	0.0004311	ko00280
**Metabolism**	**Biosynthesis of other secondary metabolites**	**Flavonoid biosynthesis**	**20**	**0.13333333**	**0.00091814**	**ko00941**
Metabolism	Metabolism of other amino acids	beta-Alanine metabolism	23	0.12234043	0.00131165	ko00410
Metabolism	Lipid metabolism	Linoleic acid metabolism	8	0.18181818	0.00498058	ko00591
Metabolism	Carbohydrate metabolism	Butanoate metabolism	15	0.12820513	0.00554366	ko00650
Metabolism	Metabolism of terpenoids and polyketides	Limonene and pinene degradation	12	0.13793103	0.00701069	ko00903
Metabolism	Carbohydrate metabolism	Pyruvate metabolism	48	0.08839779	0.00763093	ko00620
Metabolism	Carbohydrate metabolism	Glycolysis / Gluconeogenesis	55	0.08540373	0.00888767	ko00010
Metabolism	Carbohydrate metabolism	Amino sugar and nucleotide sugar metabolism	31	0.09509202	0.01110636	ko00520
Metabolism	Carbohydrate metabolism	Ascorbate and aldarate metabolism	28	0.09722222	0.01159447	ko00053
Metabolism	Amino acid metabolism	Alanine, aspartate and glutamate metabolism	25	0.09960159	0.01242808	ko00250
Metabolism	Amino acid metabolism	Lysine degradation	19	0.10734463	0.01319823	ko00310
Metabolism	Amino acid metabolism	Arginine and proline metabolism	26	0.09774436	0.01371978	ko00330
Metabolism	Carbohydrate metabolism	Propanoate metabolism	16	0.11267606	0.01429239	ko00640

Supplementary Table 8 KEGG enrichment result for genes in cluster 4 (*P*<0.05 and top 20 pathways).

KEGG A class	KEGG B class	Pathway	Count	Rich factor	*P* value	Pathway ID
**Cellular Processes**	**Transport and catabolism**	**Peroxisome**	**66**	**0.17886179**	**1.65e-09**	**ko04146**
Metabolism	Lipid metabolism	Fatty acid degradation	54	0.18367347	2.00e-08	ko00071
Metabolism	Carbohydrate metabolism	Glyoxylate and dicarboxylate metabolism	71	0.15536105	1.62e-07	ko00630
Metabolism	Amino acid metabolism	Valine, leucine and isoleucine degradation	45	0.17045455	2.56e-06	ko00280
Metabolism	Amino acid metabolism	Tryptophan metabolism	47	0.15932203	1.02e-05	ko00380
Metabolism	Carbohydrate metabolism	Pyruvate metabolism	74	0.13627993	1.33e-05	ko00620
Metabolism	Metabolism of terpenoids and polyketides	Limonene and pinene degradation	20	0.22988506	2.21e-05	ko00903
Metabolism	Metabolism of cofactors and vitamins	Pantothenate and CoA biosynthesis	29	0.18238994	4.25e-05	ko00770
Metabolism	Lipid metabolism	Glycerolipid metabolism	45	0.14802632	9.73e-05	ko00561
Metabolism	Metabolism of terpenoids and polyketides	Diterpenoid biosynthesis	15	0.23076923	0.00021889	ko00904
Metabolism	Amino acid metabolism	Histidine metabolism	21	0.18421053	0.0004041	ko00340
Metabolism	Global and overview maps	Fatty acid metabolism	49	0.13351499	0.00059602	ko01212
Metabolism	Global and overview maps	Biosynthesis of secondary metabolites	374	0.0953352	0.00065588	ko01110
Metabolism	Metabolism of other amino acids	beta-Alanine metabolism	29	0.15425532	0.0008241	ko00410
Metabolism	Carbohydrate metabolism	Ascorbate and aldarate metabolism	40	0.13888889	0.0008548	ko00053
Metabolism	Global and overview maps	Carbon metabolism	127	0.10672269	0.00156468	ko01200
Metabolism	Lipid metabolism	Cutin, suberine and wax biosynthesis	13	0.19402985	0.00305294	ko00073
Metabolism	Metabolism of terpenoids and polyketides	Sesquiterpenoid and triterpenoid biosynthesis	7	0.28	0.00336401	ko00909
Metabolism	Lipid metabolism	Sphingolipid metabolism	24	0.14906832	0.00351117	ko00600
Metabolism	Global and overview maps	Metabolic pathways	627	0.08934169	0.00398339	ko01100

Supplementary Table 9 KEGG enrichment result for genes in cluster 5 (P<0.05 and top 20 pathways).

KEGG A class	KEGG B class	Pathway	Count	Rich factor	*P* value	Pathway ID
Metabolism	Biosynthesis of other secondary metabolites	Indole alkaloid biosynthesis	5	0.83333333	2.81e-06	ko00901
**Organismal Systems**	**Environmental adaptation**	**Plant-pathogen interaction**	**70**	**0.09067358**	**2.12e-05**	**ko04626**
**Metabolism**	**Amino acid metabolism**	**Phenylalanine metabolism**	**15**	**0.15306122**	**0.00027693**	**ko00360**
Metabolism	Biosynthesis of other secondary metabolites	Betalain biosynthesis	8	0.21621622	0.00073859	ko00965
**Environmental Information Processing**	**Signal transduction**	**Plant hormone signal transduction**	**35**	**0.0951087**	**0.00108385**	**ko04075**
Metabolism	Biosynthesis of other secondary metabolites	Isoquinoline alkaloid biosynthesis	10	0.17241379	0.00110711	ko00950
Metabolism	Lipid metabolism	Arachidonic acid metabolism	11	0.15714286	0.0014068	ko00590
Metabolism	Nucleotide metabolism	Pyrimidine metabolism	31	0.0922619	0.00323883	ko00240
Metabolism	Amino acid metabolism	Tyrosine metabolism	17	0.09883721	0.01356482	ko00350
Genetic Information Processing	Transcription	RNA polymerase	14	0.10447761	0.01527661	ko03020
Metabolism	Glycan biosynthesis and metabolism	Other types of O-glycan biosynthesis	7	0.14285714	0.01674526	ko00514
**Environmental Information Processing**	**Signal transduction**	**MAPK signaling pathway - plant**	**41**	**0.07692308**	**0.01800128**	**ko04016**
Metabolism	Metabolism of cofactors and vitamins	Vitamin B6 metabolism	6	0.13953488	0.02883028	ko00750
Metabolism	Glycan biosynthesis and metabolism	Glycosphingolipid biosynthesis - globo and isoglobo series	5	0.14285714	0.04068084	ko00603
Genetic Information Processing	Replication and repair	Non-homologous end-joining	3	0.2	0.04566475	ko03450

Supplementary Table 10 KEGG enrichment result for genes in cluster 6 (P<0.05).

KEGG A class	KEGG B class	Pathway	Count	Rich factor	*P* value	Pathway ID
Genetic Information Processing	Translation	Ribosome	380	0.21064302	8.05e-38	ko03010
**Metabolism**	**Energy metabolism**	**Photosynthesis**	**34**	**0.28099174**	**3.29e-07**	**ko00195**
**Metabolism**	**Energy metabolism**	**Carbon fixation in photosynthetic organisms**	**58**	**0.17791411**	**0.00031286**	**ko00710**
Metabolism	Metabolism of cofactors and vitamins	Lipoic acid metabolism	5	0.55555556	0.00157794	ko00785
Metabolism	Global and overview maps	Carbon metabolism	167	0.14033613	0.00161054	ko01200
Cellular Processes	Transport and catabolism	Phagosome	56	0.16519174	0.00236345	ko04145
**Metabolism**	**Energy metabolism**	**Photosynthesis - antenna proteins**	**14**	**0.24561404**	**0.0037987**	**ko00196**
Metabolism	Global and overview maps	Biosynthesis of amino acids	119	0.14251497	0.00446287	ko01230
Metabolism	Carbohydrate metabolism	Pentose phosphate pathway	41	0.16269841	0.01081549	ko00030
Metabolism	Amino acid metabolism	Valine, leucine and isoleucine biosynthesis	13	0.21666667	0.01531097	ko00290
Genetic Information Processing	Folding, sorting and degradation	Protein processing in endoplasmic reticulum	122	0.13347921	0.02850271	ko04141
Metabolism	Energy metabolism	Oxidative phosphorylation	72	0.13899614	0.0380422	ko00190

Supplementary Table 11 KEGG enrichment result for genes in cluster 7 (*P*<0.05).

KEGG A class	KEGG B class	Pathway	Count	Rich factor	*P* value	Pathway ID
**Metabolism**	**Amino acid metabolism**	**Phenylalanine, tyrosine and tryptophan biosynthesis**	**19**	**0.18446602**	**0.00022836**	**ko00400**
Metabolism	Global and overview maps	Metabolic pathways	582	0.08292961	0.00066242	ko01100
Metabolism	Lipid metabolism	Glycerophospholipid metabolism	33	0.13414634	0.00089247	ko00564
Metabolism	Global and overview maps	Biosynthesis of amino acids	84	0.1005988	0.00390381	ko01230
Metabolism	Global and overview maps	Biosynthesis of secondary metabolites	330	0.0841193	0.01000423	ko01110
Metabolism	Energy metabolism	Nitrogen metabolism	16	0.13913043	0.01256162	ko00910
Metabolism	Amino acid metabolism	Lysine biosynthesis	5	0.25	0.01449437	ko00300
Metabolism	Glycan biosynthesis and metabolism	Glycosylphosphatidylinositol (GPI)-anchor biosynthesis	8	0.17777778	0.01813668	ko00563
Metabolism	Nucleotide metabolism	Pyrimidine metabolism	36	0.10714286	0.02099485	ko00240
Metabolism	Metabolism of terpenoids and polyketides	Diterpenoid biosynthesis	10	0.15384615	0.02314248	ko00904
Metabolism	Lipid metabolism	Cutin, suberine and wax biosynthesis	10	0.14925373	0.02801609	ko00073
Metabolism	Metabolism of other amino acids	Glutathione metabolism	30	0.10714286	0.03286152	ko00480

Supplementary Table 12 KEGG enrichment result for genes in cluster 8 (P<0.05).

KEGG A class	KEGG B class	Pathway	Count	Rich factor	*P* value	Pathway ID
Environmental Information Processing	Membrane transport	ABC transporters	22	0.10891089	6.02e-06	ko02010
Genetic Information Processing	Transcription	RNA polymerase	16	0.11940299	3.61e-05	ko03020
Metabolism	Biosynthesis of other secondary metabolites	Betalain biosynthesis	6	0.16216216	0.00219689	ko00965
Metabolism	Carbohydrate metabolism	Pentose and glucuronate interconversions	20	0.06756757	0.00722891	ko00040
**Organismal Systems**	**Environmental adaptation**	**Plant-pathogen interaction**	**41**	**0.05310881**	**0.01290324**	**ko04626**
Genetic Information Processing	Translation	Nucleocytoplasmic transport	21	0.06017192	0.02023836	ko03013

Supplementary Table 13 KEGG enrichment result for genes in cluster 9 (*P*<0.05).

KEGG A class	KEGG B class	Pathway	Count	Rich factor	*P* value	Rich factor
Metabolism	Energy metabolism	Oxidative phosphorylation	78	0.15057915	1.50e-06	ko00190
Metabolism	Metabolism of cofactors and vitamins	One carbon pool by folate	16	0.26666667	4.16e-05	ko00670
Metabolism	Amino acid metabolism	Alanine, aspartate and glutamate metabolism	40	0.15936255	0.00015842	ko00250
Metabolism	Global and overview maps	Carbon metabolism	139	0.11680672	0.00023714	ko01200
**Genetic Information Processing**	**Folding, sorting and degradation**	**Proteasome**	**32**	**0.15458937**	**0.00116412**	**ko03050**
Metabolism	Biosynthesis of other secondary metabolites	Aflatoxin biosynthesis	7	0.31818182	0.00208172	ko00254
Metabolism	Global and overview maps	Metabolic pathways	663	0.09447136	0.00381405	ko01100
Metabolism	Carbohydrate metabolism	Pentose phosphate pathway	35	0.13888889	0.00446656	ko00030
Genetic Information Processing	Transcription	Spliceosome	80	0.11695906	0.00478688	ko03040
Metabolism	Global and overview maps	Biosynthesis of amino acids	95	0.11377246	0.00496489	ko01230
Metabolism	Nucleotide metabolism	Purine metabolism	32	0.13852814	0.00660627	ko00230
Metabolism	Carbohydrate metabolism	Glycolysis / Gluconeogenesis	75	0.11645963	0.00684981	ko00010
Metabolism	Carbohydrate metabolism	Butanoate metabolism	18	0.15384615	0.01343399	ko00650
Metabolism	Global and overview maps	Biosynthesis of secondary metabolites	376	0.09584502	0.02187981	ko01110
Metabolism	Carbohydrate metabolism	Amino sugar and nucleotide sugar metabolism	39	0.1196319	0.02947883	ko00520
Genetic Information Processing	Folding, sorting and degradation	Protein processing in endoplasmic reticulum	96	0.10503282	0.03573895	ko04141
Metabolism	Carbohydrate metabolism	C5-Branched dibasic acid metabolism	5	0.22727273	0.038728	ko00660
Metabolism	Carbohydrate metabolism	Ascorbate and aldarate metabolism	34	0.11805556	0.04678626	ko00053

Supplementary Table 14 KEGG enrichment result for genes in cluster 10 (P<0.05 and top 20 pathways).

KEGG A class	KEGG B class	Pathway	Count	Rich factor	*P* value	Pathway ID
Genetic Information Processing	Translation	Ribosome	444	0.24611973	1.95e-33	ko03010
Metabolism	Carbohydrate metabolism	Citrate cycle (TCA cycle)	103	0.28690808	2.90e-12	ko00020
Metabolism	Global and overview maps	Fatty acid metabolism	92	0.2506812	7.12e-08	ko01212
Metabolism	Global and overview maps	Carbon metabolism	233	0.19579832	6.90e-07	ko01200
Metabolism	Lipid metabolism	Fatty acid biosynthesis	46	0.28571429	3.46e-06	ko00061
**Metabolism**	**Energy metabolism**	**Carbon fixation in photosynthetic organisms**	**76**	**0.23312883**	**1.67e-05**	**ko00710**
Metabolism	Global and overview maps	Biosynthesis of amino acids	165	0.19760479	1.91e-05	ko01230
Metabolism	Global and overview maps	Biosynthesis of secondary metabolites	640	0.16314045	0.00028969	ko01110
Metabolism	Lipid metabolism	Biosynthesis of unsaturated fatty acids	42	0.24561404	0.00038862	ko01040
Metabolism	Biosynthesis of other secondary metabolites	Aflatoxin biosynthesis	10	0.45454545	0.00052309	ko00254
Metabolism	Carbohydrate metabolism	Pyruvate metabolism	106	0.19521179	0.00090658	ko00620
Metabolism	Carbohydrate metabolism	Pentose phosphate pathway	55	0.21825397	0.00125631	ko00030
Metabolism	Carbohydrate metabolism	Glycolysis / Gluconeogenesis	119	0.18478261	0.0034512	ko00010
Metabolism	Amino acid metabolism	Cysteine and methionine metabolism	79	0.19554455	0.0036671	ko00270
Metabolism	Lipid metabolism	Steroid biosynthesis	24	0.25531915	0.00376194	ko00100
Metabolism	Global and overview maps	2-Oxocarboxylic acid metabolism	51	0.20987654	0.00431062	ko01210
Metabolism	Metabolism of cofactors and vitamins	Biotin metabolism	19	0.25675676	0.00873302	ko00780
Metabolism	Amino acid metabolism	Alanine, aspartate and glutamate metabolism	50	0.19920319	0.01286527	ko00250
Metabolism	Carbohydrate metabolism	Glyoxylate and dicarboxylate metabolism	82	0.17943107	0.02648959	ko00630
Metabolism	Metabolism of other amino acids	Selenocompound metabolism	22	0.20952381	0.04908654	ko00450

Supplementary Table 15 KEGG enrichment result for genes in cluster 11 (P<0.05 and top 20 pathways).

KEGG A class	KEGG B class	Pathway	Count	Rich factor	*P* value	Pathway ID
Metabolism	Nucleotide metabolism	Pyrimidine metabolism	44	0.13095238	3.79e-05	ko00240
Genetic Information Processing	Replication and repair	Base excision repair	15	0.2027027	0.00014927	ko03410
Genetic Information Processing	Replication and repair	Mismatch repair	16	0.19277108	0.00016906	ko03430
Genetic Information Processing	Replication and repair	Homologous recombination	17	0.13709677	0.00552875	ko03440
Genetic Information Processing	Replication and repair	DNA replication	15	0.14018692	0.00720989	ko03030
Metabolism	Carbohydrate metabolism	Starch and sucrose metabolism	51	0.09770115	0.00891761	ko00500
Metabolism	Glycan biosynthesis and metabolism	Glycosphingolipid biosynthesis - globo and isoglobo series	7	0.2	0.00940337	ko00603
**Organismal Systems**	**Environmental adaptation**	**Plant-pathogen interaction**	**71**	**0.09196891**	**0.00955459**	**ko04626**
Genetic Information Processing	Replication and repair	Nucleotide excision repair	17	0.12592593	0.0126579	ko03420
Environmental Information Processing	Membrane transport	ABC transporters	23	0.11386139	0.01369149	ko02010
Metabolism	Metabolism of other amino acids	Cyanoamino acid metabolism	11	0.13924051	0.02066359	ko00460
Genetic Information Processing	Translation	mRNA surveillance pathway	34	0.09912536	0.02393794	ko03015
Metabolism	Carbohydrate metabolism	Pentose and glucuronate interconversions	30	0.10135135	0.02492433	ko00040
Metabolism	Glycan biosynthesis and metabolism	Various types of N-glycan biosynthesis	12	0.13043478	0.02572597	ko00513
Metabolism	Metabolism of cofactors and vitamins	Folate biosynthesis	8	0.1509434	0.02932774	ko00790
Genetic Information Processing	Translation	Nucleocytoplasmic transport	34	0.0974212	0.02985963	ko03013
Metabolism	Lipid metabolism	Linoleic acid metabolism	7	0.15909091	0.03119512	ko00591
Metabolism	Glycan biosynthesis and metabolism	N-Glycan biosynthesis	14	0.11965812	0.03289107	ko00510
Genetic Information Processing	Transcription	Spliceosome	60	0.0877193	0.03779115	ko03040
Genetic Information Processing	Folding, sorting and degradation	RNA degradation	30	0.09615385	0.04557379	ko03018

Supplementary Table 16 KEGG enrichment result for genes in cluster 12 (P<0.05).

KEGG A class	KEGG B class	Pathway	Count	Rich factor	*P* value	Pathway ID
**Environmental Information Processing**	**Signal transduction**	**Plant hormone signal transduction**	**44**	**0.11956522**	**6.10e-06**	**ko04075**
Cellular Processes	Transport and catabolism	Phagosome	34	0.10029499	0.00172841	ko04145
Metabolism	Metabolism of other amino acids	Cyanoamino acid metabolism	10	0.12658228	0.01737071	ko00460
Metabolism	Energy metabolism	Nitrogen metabolism	13	0.11304348	0.01787061	ko00910
Genetic Information Processing	Translation	mRNA surveillance pathway	30	0.08746356	0.0200098	ko03015
**Organismal Systems**	**Environmental adaptation**	**Plant-pathogen interaction**	**59**	**0.07642487**	**0.0239312**	**ko04626**
Genetic Information Processing	Translation	Nucleocytoplasmic transport	30	0.08595989	0.02467956	ko03013
Metabolism	Biosynthesis of other secondary metabolites	Biosynthesis of various plant secondary metabolites	11	0.1122449	0.0292824	ko00999
**Environmental Information Processing**	**Signal transduction**	**MAPK signaling pathway - plant**	**42**	**0.07879925**	**0.03369262**	**ko04016**
Metabolism	Metabolism of cofactors and vitamins	Vitamin B6 metabolism	6	0.13953488	0.03909259	ko00750
Genetic Information Processing	Translation	Aminoacyl-tRNA biosynthesis	15	0.09433962	0.04858674	ko00970

Supplementary Table 17 The number of differentially expressed genes.

Sample	DEGs	Up-related	Down-related
3 dpi	4679	2531	2148
5 dpi	6962	3280	3682
7 dpi	6098	3068	3030
9 dpi	8100	3981	4119

Supplementary Table 18 KEGG enrichment result for genes up-regulated at 3 d (*P*<0.05 and top 20 pathways).

KEGG A class	KEGG B class	Pathway	Count	Rich factor	*P* value	Pathway ID
Organismal Systems	Environmental adaptation	Plant-pathogen interaction	91	0.11787565	6.55e-14	ko04626
Environmental Information Processing	Signal transduction	Plant hormone signal transduction	45	0.12228261	6.42e-08	ko04075
Metabolism	Global and overview maps	Metabolic pathways	433	0.06169849	8.93e-08	ko01100
**Metabolism**	**Biosynthesis of other secondary metabolites**	**Flavonoid biosynthesis**	**25**	**0.16666667**	**1.89e-07**	**ko00941**
Metabolism	Biosynthesis of other secondary metabolites	Phenylpropanoid biosynthesis	33	0.12692308	1.62e-06	ko00940
Metabolism	Carbohydrate metabolism	Starch and sucrose metabolism	50	0.09578544	1.79e-05	ko00500
Organismal Systems	Environmental adaptation	Circadian rhythm - plant	17	0.16346154	2.21e-05	ko04712
Metabolism	Biosynthesis of other secondary metabolites	Biosynthesis of various plant secondary metabolites	16	0.16326531	3.88e-05	ko00999
Metabolism	Carbohydrate metabolism	Pentose and glucuronate interconversions	32	0.10810811	6.25e-05	ko00040
Metabolism	Global and overview maps	Biosynthesis of secondary metabolites	248	0.06321693	0.00010099	ko01110
Environmental Information Processing	Signal transduction	MAPK signaling pathway - plant	48	0.09005629	0.00012189	ko04016
Metabolism	Carbohydrate metabolism	Amino sugar and nucleotide sugar metabolism	32	0.09815951	0.00037133	ko00520
Metabolism	Metabolism of cofactors and vitamins	Ubiquinone and other terpenoid-quinone biosynthesis	13	0.15116279	0.00044223	ko00130
**Metabolism**	**Lipid metabolism**	**Linoleic acid metabolism**	**8**	**0.18181818**	**0.0016471**	**ko00591**
Metabolism	Glycan biosynthesis and metabolism	Glycosphingolipid biosynthesis - globo and isoglobo series	7	0.2	0.00179752	ko00603
Metabolism	Biosynthesis of other secondary metabolites	Indole alkaloid biosynthesis	3	0.5	0.00243637	ko00901
Genetic Information Processing	Replication and repair	DNA replication	13	0.12149533	0.00341564	ko03030
Metabolism	Metabolism of other amino acids	Taurine and hypotaurine metabolism	7	0.17948718	0.00343787	ko00430
Metabolism	Carbohydrate metabolism	Galactose metabolism	24	0.09302326	0.00386343	ko00052
Metabolism	Metabolism of other amino acids	Cyanoamino acid metabolism	10	0.12658228	0.00726082	ko00460

Supplementary Table 19 KEGG enrichment result for genes down-regulated at 3 d (P<0.05 and top 20 pathways).

KEGG A class	KEGG B class	Pathway	Count	Rich factor	*P* value	Pathway ID
**Metabolism**	**Energy metabolism**	**Photosynthesis**	**30**	**0.24793388**	**9.09e-15**	**ko00195**
**Metabolism**	**Metabolism of terpenoids and polyketides**	**Carotenoid biosynthesis**	**23**	**0.21904762**	**1.86e-10**	**ko00906**
Metabolism	Global and overview maps	Biosynthesis of secondary metabolites	244	0.0621973	2.06e-09	ko01110
Metabolism	Global and overview maps	Metabolic pathways	389	0.0554289	2.47e-09	ko01100
Metabolism	Metabolism of terpenoids and polyketides	Terpenoid backbone biosynthesis	19	0.16521739	8.02e-07	ko00900
Metabolism	Biosynthesis of other secondary metabolites	Flavonoid biosynthesis	21	0.14	3.47e-06	ko00941
Metabolism	Metabolism of cofactors and vitamins	Ubiquinone and other terpenoid-quinone biosynthesis	14	0.1627907	2.66e-05	ko00130
Metabolism	Metabolism of terpenoids and polyketides	Diterpenoid biosynthesis	12	0.18461538	2.73e-05	ko00904
**Metabolism**	**Biosynthesis of other secondary metabolites**	**Flavone and flavonol biosynthesis**	**9**	**0.24324324**	**2.81e-05**	**ko00944**
Metabolism	Metabolism of cofactors and vitamins	Porphyrin metabolism	16	0.14035088	4.82e-05	ko00860
Environmental Information Processing	Signal transduction	Plant hormone signal transduction	34	0.0923913	5.46e-05	ko04075
Metabolism	Metabolism of terpenoids and polyketides	Sesquiterpenoid and triterpenoid biosynthesis	6	0.24	0.0006796	ko00909
Metabolism	Energy metabolism	Photosynthesis - antenna proteins	9	0.15789474	0.00091093	ko00196
Metabolism	Metabolism of cofactors and vitamins	Thiamine metabolism	8	0.17021277	0.00104973	ko00730
**Metabolism**	**Biosynthesis of other secondary metabolites**	**Stilbenoid, diarylheptanoid and gingerol biosynthesis**	**6**	**0.21428571**	**0.00128909**	**ko00945**
**Metabolism**	**Metabolism of terpenoids and polyketides**	**Monoterpenoid biosynthesis**	**4**	**0.30769231**	**0.00207622**	**ko00902**
Metabolism	Lipid metabolism	Cutin, suberine and wax biosynthesis	9	0.13432836	0.00291007	ko00073
Metabolism	Biosynthesis of other secondary metabolites	Phenylpropanoid biosynthesis	22	0.08461538	0.00329509	ko00940
Metabolism	Carbohydrate metabolism	Inositol phosphate metabolism	13	0.08965517	0.01352492	ko00562
Metabolism	Carbohydrate metabolism	Fructose and mannose metabolism	15	0.08287293	0.01639129	ko00051

Supplementary Table 20 KEGG enrichment result for genes up-regulated at 5 d (P<0.05 and top 20 pathways).

KEGG A class	KEGG B class	Pathway	Count	Rich factor	*P* value	Pathway ID
**Metabolism**	**Biosynthesis of other secondary metabolites**	**Flavonoid biosynthesis**	**47**	**0.31333333**	**1.73e-17**	**ko00941**
Metabolism	Global and overview maps	Metabolic pathways	636	0.09062411	1.79e-09	ko01100
Metabolism	Global and overview maps	Biosynthesis of secondary metabolites	372	0.09482539	8.80e-07	ko01110
Metabolism	Biosynthesis of other secondary metabolites	Phenylpropanoid biosynthesis	41	0.15769231	8.07e-06	ko00940
Metabolism	Amino acid metabolism	Phenylalanine, tyrosine and tryptophan biosynthesis	22	0.21359223	9.26e-06	ko00400
Organismal Systems	Environmental adaptation	Plant-pathogen interaction	90	0.11658031	4.06e-05	ko04626
**Metabolism**	**Biosynthesis of other secondary metabolites**	**Flavone and flavonol biosynthesis**	**11**	**0.2972973**	**7.02e-05**	**ko00944**
Environmental Information Processing	Signal transduction	Plant hormone signal transduction	48	0.13043478	0.00021244	ko04075
Metabolism	Carbohydrate metabolism	Fructose and mannose metabolism	28	0.15469613	0.00029287	ko00051
Organismal Systems	Environmental adaptation	Circadian rhythm - plant	19	0.18269231	0.000329	ko04712
Metabolism	Global and overview maps	Biosynthesis of amino acids	89	0.10658683	0.00094181	ko01230
Metabolism	Carbohydrate metabolism	Amino sugar and nucleotide sugar metabolism	41	0.12576687	0.00122879	ko00520
Metabolism	Lipid metabolism	alpha-Linolenic acid metabolism	25	0.14619883	0.0013872	ko00592
Metabolism	Metabolism of cofactors and vitamins	Ubiquinone and other terpenoid-quinone biosynthesis	14	0.1627907	0.0057285	ko00130
Metabolism	Carbohydrate metabolism	Pentose and glucuronate interconversions	35	0.11824324	0.00719699	ko00040
Metabolism	Biosynthesis of other secondary metabolites	Biosynthesis of various plant secondary metabolites	15	0.15306122	0.00772685	ko00999
Metabolism	Biosynthesis of other secondary metabolites	Tropane, piperidine and pyridine alkaloid biosynthesis	14	0.15730337	0.00777904	ko00960
Metabolism	Metabolism of cofactors and vitamins	Nicotinate and nicotinamide metabolism	11	0.17460317	0.00803591	ko00760
Metabolism	Lipid metabolism	Glycerophospholipid metabolism	29	0.11788618	0.01407263	ko00564
Metabolism	Glycan biosynthesis and metabolism	Glycosaminoglycan degradation	9	0.17307692	0.01665029	ko00531

Supplementary Table 21 KEGG enrichment result for genes down-regulated at 5 d (P<0.05 and top 20 pathways).

KEGG A class	KEGG B class	Pathway	Count	Rich factor	*P* value	Pathway ID
**Metabolism**	**Metabolism of terpenoids and polyketides**	**Carotenoid biosynthesis**	**26**	**0.24761905**	**1.89e-09**	**ko00906**
**Metabolism**	**Energy metabolism**	**Photosynthesis**	**28**	**0.23140496**	**2.38e-09**	**ko00195**
Environmental Information Processing	Signal transduction	Plant hormone signal transduction	49	0.13315217	1.12e-06	ko04075
Metabolism	Global and overview maps	Biosynthesis of secondary metabolites	313	0.07978588	6.72e-06	ko01110
**Metabolism**	**Biosynthesis of other secondary metabolites**	**Flavone and flavonol biosynthesis**	**11**	**0.2972973**	**1.41e-05**	**ko00944**
Metabolism	Metabolism of terpenoids and polyketides	Diterpenoid biosynthesis	14	0.21538462	5.61e-05	ko00904
Metabolism	Global and overview maps	Metabolic pathways	511	0.07281277	8.28e-05	ko01100
Metabolism	Metabolism of cofactors and vitamins	Porphyrin metabolism	19	0.16666667	0.00012322	ko00860
Metabolism	Energy metabolism	Photosynthesis - antenna proteins	11	0.19298246	0.0009276	ko00196
Metabolism	Metabolism of cofactors and vitamins	Ubiquinone and other terpenoid-quinone biosynthesis	14	0.1627907	0.00117662	ko00130
Metabolism	Carbohydrate metabolism	Inositol phosphate metabolism	19	0.13103448	0.00255827	ko00562
Metabolism	Metabolism of terpenoids and polyketides	Terpenoid backbone biosynthesis	16	0.13913043	0.0029573	ko00900
Metabolism	Lipid metabolism	Cutin, suberine and wax biosynthesis	11	0.1641791	0.00358274	ko00073
Environmental Information Processing	Signal transduction	Phosphatidylinositol signaling system	17	0.12318841	0.00780243	ko04070
Metabolism	Lipid metabolism	Fatty acid elongation	9	0.15789474	0.01041638	ko00062
Organismal Systems	Environmental adaptation	Circadian rhythm - plant	13	0.125	0.01657062	ko04712
Metabolism	Glycan biosynthesis and metabolism	Glycosaminoglycan degradation	8	0.15384615	0.01776972	ko00531
Metabolism	Metabolism of terpenoids and polyketides	Brassinosteroid biosynthesis	6	0.18181818	0.01778594	ko00905
Metabolism	Metabolism of cofactors and vitamins	Riboflavin metabolism	6	0.17142857	0.02337124	ko00740
Metabolism	Metabolism of other amino acids	Glutathione metabolism	27	0.09642857	0.02468304	ko00480

Supplementary Table 22 KEGG enrichment result for genes up-regulated at 7 d (*P*<0.05 and top 20 pathways).

KEGG A class	KEGG B class	Pathway	Count	Rich factor	*P* value	Pathway ID
Metabolism	Global and overview maps	Metabolic pathways	603	0.08592192	3.70e-12	ko01100
Organismal Systems	Environmental adaptation	Plant-pathogen interaction	106	0.1373057	1.58e-11	ko04626
**Metabolism**	**Biosynthesis of other secondary metabolites**	**Flavonoid biosynthesis**	**35**	**0.23333333**	**2.11e-10**	**ko00941**
Metabolism	Global and overview maps	Biosynthesis of secondary metabolites	353	0.08998216	4.34e-08	ko01110
Environmental Information Processing	Signal transduction	Plant hormone signal transduction	55	0.14945652	9.04e-08	ko04075
Organismal Systems	Environmental adaptation	Circadian rhythm - plant	24	0.23076923	1.81e-07	ko04712
Metabolism	Biosynthesis of other secondary metabolites	Phenylpropanoid biosynthesis	42	0.16153846	3.69e-07	ko00940
Environmental Information Processing	Signal transduction	MAPK signaling pathway - plant	62	0.1163227	6.70e-05	ko04016
Metabolism	Metabolism of cofactors and vitamins	Nicotinate and nicotinamide metabolism	14	0.22222222	0.00010053	ko00760
**Metabolism**	**Biosynthesis of other secondary metabolites**	**Flavone and flavonol biosynthesis**	**10**	**0.27027027**	**0.00017786**	**ko00944**
Metabolism	Lipid metabolism	alpha-Linolenic acid metabolism	25	0.14619883	0.00040797	ko00592
Metabolism	Amino acid metabolism	Phenylalanine, tyrosine and tryptophan biosynthesis	17	0.16504854	0.00084436	ko00400
Metabolism	Carbohydrate metabolism	Amino sugar and nucleotide sugar metabolism	39	0.1196319	0.00084954	ko00520
Environmental Information Processing	Signal transduction	Phosphatidylinositol signaling system	20	0.14492754	0.00165402	ko04070
Metabolism	Global and overview maps	Biosynthesis of amino acids	80	0.09580838	0.00304832	ko01230
Metabolism	Lipid metabolism	Linoleic acid metabolism	9	0.20454545	0.0031657	ko00591
Metabolism	Biosynthesis of other secondary metabolites	Biosynthesis of various plant secondary metabolites	15	0.15306122	0.00355085	ko00999
Metabolism	Carbohydrate metabolism	Glycolysis / Gluconeogenesis	63	0.09782609	0.00515113	ko00010
Metabolism	Metabolism of cofactors and vitamins	Ubiquinone and other terpenoid-quinone biosynthesis	13	0.15116279	0.007115	ko00130
Metabolism	Carbohydrate metabolism	Fructose and mannose metabolism	22	0.12154696	0.00883691	ko00051

Supplementary Table 23 KEGG enrichment result for genes down-regulated at 7 d (*P*<0.05).

KEGG A class	KEGG B class	Pathway	Count	Rich factor	*P* value	Pathway ID
Environmental Information Processing	Signal transduction	Plant hormone signal transduction	45	0.12228261	1.36e-08	ko04075
**Metabolism**	**Metabolism of terpenoids and polyketides**	**Carotenoid biosynthesis**	**20**	**0.19047619**	**1.51e-07**	**ko00906**
Genetic Information Processing	Replication and repair	Base excision repair	16	0.21621622	4.37e-07	ko03410
Metabolism	Metabolism of cofactors and vitamins	Porphyrin metabolism	17	0.14912281	3.85e-05	ko00860
Metabolism	Carbohydrate metabolism	Starch and sucrose metabolism	47	0.09003831	4.10e-05	ko00500
**Genetic Information Processing**	**Replication and repair**	**Non-homologous end-joining**	**5**	**0.33333333**	**0.00055395**	**ko03450**
Metabolism	Metabolism of terpenoids and polyketides	Brassinosteroid biosynthesis	7	0.21212121	0.00091948	ko00905
Genetic Information Processing	Replication and repair	Mismatch repair	11	0.13253012	0.00233488	ko03430
Metabolism	Metabolism of terpenoids and polyketides	Diterpenoid biosynthesis	9	0.13846154	0.00422343	ko00904
Genetic Information Processing	Replication and repair	Homologous recombination	13	0.10483871	0.00773116	ko03440
Organismal Systems	Environmental adaptation	Circadian rhythm - plant	11	0.10576923	0.01283756	ko04712
Genetic Information Processing	Replication and repair	Nucleotide excision repair	13	0.0962963	0.01516009	ko03420
Metabolism	Metabolism of terpenoids and polyketides	Terpenoid backbone biosynthesis	11	0.09565217	0.02537874	ko00900
Metabolism	Carbohydrate metabolism	Inositol phosphate metabolism	13	0.08965517	0.02579187	ko00562
**Metabolism**	**Biosynthesis of other secondary metabolites**	**Indole alkaloid biosynthesis**	**2**	**0.33333333**	**0.03151651**	**ko00901**
Metabolism	Lipid metabolism	Cutin, suberine and wax biosynthesis	7	0.10447761	0.04499195	ko00073

Supplementary Table 24 KEGG enrichment result for genes up-regulated at 9 d (*P*<0.05 and top 20 pathways).

KEGG A class	KEGG B class	Pathway	Count	Rich factor	*P* value	Pathway ID
Metabolism	Global and overview maps	Metabolic pathways	758	0.10800798	2.71e-21	ko01100
**Metabolism**	**Biosynthesis of other secondary metabolites**	**Flavonoid biosynthesis**	**43**	**0.28666667**	**5.03e-13**	**ko00941**
Metabolism	Global and overview maps	Biosynthesis of secondary metabolites	433	0.11037471	8.24e-11	ko01110
**Metabolism**	**Biosynthesis of other secondary metabolites**	**Flavone and flavonol biosynthesis**	**13**	**0.35135135**	**5.99e-06**	**ko00944**
Metabolism	Lipid metabolism	alpha-Linolenic acid metabolism	33	0.19298246	6.65e-06	ko00592
Environmental Information Processing	Signal transduction	Plant hormone signal transduction	57	0.1548913	6.82e-06	ko04075
Metabolism	Carbohydrate metabolism	Amino sugar and nucleotide sugar metabolism	52	0.1595092	7.37e-06	ko00520
Metabolism	Biosynthesis of other secondary metabolites	Phenylpropanoid biosynthesis	42	0.16153846	4.02e-05	ko00940
Metabolism	Carbohydrate metabolism	Fructose and mannose metabolism	30	0.16574586	0.00030855	ko00051
Organismal Systems	Environmental adaptation	Circadian rhythm - plant	20	0.19230769	0.00043667	ko04712
Organismal Systems	Environmental adaptation	Plant-pathogen interaction	91	0.11787565	0.00085976	ko04626
Metabolism	Global and overview maps	Biosynthesis of amino acids	97	0.11616766	0.00095057	ko01230
Metabolism	Lipid metabolism	Glycerolipid metabolism	42	0.13815789	0.00122512	ko00561
Metabolism	Lipid metabolism	Glycerophospholipid metabolism	35	0.14227642	0.00182343	ko00564
Environmental Information Processing	Signal transduction	MAPK signaling pathway - plant	65	0.12195122	0.00201431	ko04016
Metabolism	Lipid metabolism	Arachidonic acid metabolism	14	0.2	0.00205779	ko00590
Metabolism	Metabolism of cofactors and vitamins	Nicotinate and nicotinamide metabolism	13	0.20634921	0.00219005	ko00760
Metabolism	Amino acid metabolism	Phenylalanine, tyrosine and tryptophan biosynthesis	18	0.17475728	0.00256309	ko00400
Environmental Information Processing	Signal transduction	Phosphatidylinositol signaling system	22	0.15942029	0.00305858	ko04070
Metabolism	Lipid metabolism	Linoleic acid metabolism	10	0.22727273	0.00330432	ko00591

Supplementary Table 25 KEGG enrichment result for genes down-regulated at 9 d (P<0.05).

KEGG A class	KEGG B class	Pathway	Count	Rich factor	*P* value	Pathway ID
**Metabolism**	**Energy metabolism**	**Photosynthesis**	**34**	**0.28099174**	**2.77e-10**	**ko00195**
Metabolism	Metabolism of cofactors and vitamins	Porphyrin metabolism	28	0.24561404	2.38e-07	ko00860
Genetic Information Processing	Replication and repair	Base excision repair	20	0.27027027	2.52e-06	ko03410
Environmental Information Processing	Signal transduction	Plant hormone signal transduction	54	0.14673913	6.08e-05	ko04075
Metabolism	Metabolism of terpenoids and polyketides	Carotenoid biosynthesis	22	0.20952381	6.54e-05	ko00906
**Metabolism**	**Energy metabolism**	**Photosynthesis - antenna proteins**	**14**	**0.24561404**	**0.00024224**	**ko00196**
Genetic Information Processing	Replication and repair	Non-homologous end-joining	6	0.4	0.00098623	ko03450
Organismal Systems	Environmental adaptation	Circadian rhythm - plant	19	0.18269231	0.00120602	ko04712
Metabolism	Metabolism of cofactors and vitamins	Thiamine metabolism	10	0.21276596	0.00561529	ko00730
Metabolism	Biosynthesis of other secondary metabolites	Flavonoid biosynthesis	21	0.14	0.01686103	ko00941
Metabolism	Global and overview maps	Biosynthesis of secondary metabolites	368	0.09380576	0.01761661	ko01110
Metabolism	Global and overview maps	Metabolic pathways	636	0.09062411	0.01970464	ko01100
Metabolism	Glycan biosynthesis and metabolism	Mannose type O-glycan biosynthesis	2	0.66666667	0.02069575	ko00515
Metabolism	Metabolism of cofactors and vitamins	Riboflavin metabolism	7	0.2	0.02654479	ko00740
Genetic Information Processing	Translation	Aminoacyl-tRNA biosynthesis	21	0.13207547	0.0302072	ko00970
Metabolism	Biosynthesis of other secondary metabolites	Flavone and flavonol biosynthesis	7	0.18918919	0.03505114	ko00944
Metabolism	Metabolism of terpenoids and polyketides	Terpenoid backbone biosynthesis	16	0.13913043	0.03565581	ko00900
Metabolism	Energy metabolism	Sulfur metabolism	11	0.15277778	0.04151471	ko00920

Supplementary Table 26 The 63 genes that upregulated and enriched in the flavonoid biosynthesis pathway at any time point.

No.	KEGG	Log_2fold			
		3 d	5 d	7 d	9 d
1	flavonol synthase	6.33373646	6.2559	6.0909	7.2952
2	flavonol synthase	2.85594245	-0.372	2.53806589	4.6970054
3	naringenin 3-dioxygenase	5.26429093	-1.1745561	3.89578029	2.6658829
4	shikimate O-hydroxycinnamoyltransferase	1.96175043	2.34493822	1.82795662	3.13987926
5	flavonoid 3'-monooxygenase	1.43484713	2.74625149	0.77858785	2.63212601
6	caffeoyl-CoA O-methyltransferase	1.42651975	4.49554026	2.50587274	3.30627507
7	pinosylvin synthase	3.75987618	4.52907675	6.8958798	8.45073708
8	flavonol synthase	2.25349978	4.98041291	6.58411399	4.10938906
9	flavonol synthase	1.81709586	1.03416403	1.49393924	1.99132968
10	caffeoyl-CoA O-methyltransferase	1.02042517	1.77417138	2.57677417	2.4745249
11	chalcone isomerase	1.31567339	2.95012347	2.98445381	3.17647166
12	trans-cinnamate 4-monooxygenase	3.54312385	5.15299939	2.8260056	5.57141194
13	anthocyanidin reductase	2.60449067	3.56292064	3.85340058	4.92017396
14	chalcone synthase	2.06346315	3.33964492	4.7803	7.52669485
15	5-O-(4-coumaroyl)-D-quinate 3'-monooxygenase	1.0749054	2.70194618	2.57151798	1.97862239
16	flavonol synthase	1.40246164	1.64980137	1.65019718	1.57353525
17	bifunctional dihydroflavonol 4-reductase/flavanone 4-reductase	1.77098419	-0.0605996	2.4437	2.42763677
18	5-O-(4-coumaroyl)-D-quinate 3'-monooxygenase	1.7965087	1.9581598	1.72522776	2.44275938
19	chalcone synthase	2.74556576	2.65616284	4.54686801	5.22790504
20	chalcone synthase	7.019669	7.4278722	7.21825803	8.34368135
21	bifunctional dihydroflavonol 4-reductase/flavanone 4-reductase	2.5814835	2.98296516	3.74074307	2.55904823
22	leucoanthocyanidin reductase	1.60986863	3.21111295	3.89822125	6.18807925
23	flavonol synthase	1.4938783	0.96029008	1.13918767	0.80165791
24	chalcone synthase	1.99801153	3.53204034	9.30606169	4.8715
25	flavonol synthase	3.40945097	4.9724929	6.0138641	8.1265
26	flavonol synthase	0.56569725	1.07436228	0.74745503	0.46526416
27	flavonoid 3'-monooxygenase	0.48239193	2.97700411	0.89313471	1.10137349
28	leucoanthocyanidin reductase	0.7580254	2.16617467	1.3418864	1.15558692
29	naringenin 3-dioxygenase	0.68874216	1.43978456	1.9224	0.88991669
30	bifunctional dihydroflavonol 4-reductase/flavanone 4-reductase	-0.0566915	2.43217716	0.77011672	1.48434918
31	bifunctional dihydroflavonol 4-reductase/flavanone 4-reductase	-0.8806473	1.13379719	-3.974	-1.8412697
32	flavonoid 3',5'-hydroxylase	-0.3397947	1.7363239	1.10361954	1.39636128
33	flavonol synthase	0.58752451	1.29899154	1.02642065	2.34782065
34	flavonoid 3'-monooxygenase	-1.5451139	1.04051268	-4.0985	1.80978135
35	chalcone isomerase	0.6397497	2.4807185	2.246004	2.16270869
36	naringenin 3-dioxygenase	-1.2101268	1.76085631	0.04917602	1.17825394
37	anthocyanidin reductase	4.6619	2.98941013	7.4337	3.7576
38	flavonoid 3'-monooxygenase	0.58828459	3.01576817	0.61080609	1.12715534
39	flavonol synthase	0.47600125	1.39393757	1.16315433	0.84870799
40	anthocyanidin synthase	-0.1942619	2.78040594	4.7482	4.97131327
41	bifunctional dihydroflavonol 4-reductase/flavanone 4-reductase	0.04069141	1.25522238	0.29939134	0.91294391
42	anthocyanidin reductase	-0.0942784	2.34791812	1.06096356	3.93883151

43	flavonoid 3'-monooxygenase	0.97300892	1.58141447	2.79215997	3.68123489
44	anthocyanidin reductase	-1.5344757	2.89476306	3.3269	4.6388
45	chalcone synthase	-0.5989772	1.85946924	1.87495478	2.5401855
46	anthocyanidin synthase	-1.0906069	1.24083639	0.0954	-6.098
47	anthocyanidin synthase	0.13313203	4.42793724	8.3763	7.0698
48	naringenin 3-dioxygenase	-0.3465346	5.46887858	0.52086705	2.87924802
49	anthocyanidin reductase	-0.1323794	4.19285625	-1.6884152	-0.1905339
50	trans-cinnamate 4-monooxygenase	-0.0952599	1.10674505	0.7392097	-0.5011549
51	chalcone synthase	-0.4093723	2.01380517	5.62777616	5.905708
52	anthocyanidin reductase	2.4976	2.64768788	3.09124138	3.89699603
53	bifunctional dihydroflavonol 4-reductase/flavanone 4-reductase	-1.2629557	-0.5514	2.83700549	-0.0695529
54	flavonoid 3'-monooxygenase	-0.4638587	0.06477387	1.34703787	1.2195118
55	flavonol synthase	-2.2137215	-0.0909097	1.23656986	-1.1181314
56	bifunctional dihydroflavonol 4-reductase/flavanone 4-reductase	-0.874487	-1.1240476	2.25445111	-2.9989373
57	trans-cinnamate 4-monooxygenase	-0.0620227	0.1322622	1.38081368	1.3388
58	flavonol synthase	-0.7620948	-0.3732296	0.85712894	1.46411794
59	flavonoid 3'-monooxygenase	-0.4766524	0.72728792	-3.3837	1.74415465
60	chalcone synthase	8.1386	8.6517	6.3889	7.80492736
61	flavonol synthase	-0.4397267	-0.3344851	0.43192959	1.31023943
62	5-O-(4-coumaroyl)-D-quinate 3'-monooxygenase	-1.7271847	0.34155472	0.39415778	1.00271749
63	flavonoid 3',5'-hydroxylase	-1.1550591	0.15747886	0.47695921	1.3390464

Supplementary Table 27 The result of Venn diagram of up-regulated genes enriched in the flavonoid biosynthesis pathway.

Type	Number	Gene No.
3 d&5 d&7 d&9 d	16	10;11;12;13;15;16;18;19;20;21;22;4;6;7;8;9
5 d	10	26;29;31;37;41;44;46;47;49;50
5 d&7 d&9 d	9	28;32;33;35;42;43;45;51;52
5 d&9 d	7	27;30;34;36;38;40;48
9 d	6	58;59;60;61;62;63
7 d	4	53;55;56;57
3 d&5 d	2	14;24
3 d&7 d&9 d	2	2;3
3 d&7 d	1	23
3 d	1	1
3 d&9 d	1	17
3 d&5 d&9 d	1	5
7 d&9 d	1	54
3 d&5 d&7 d	1	25
5 d&7 d	1	39

Supplementary Table 28 Statistics of identified metabolites.

Mode	All	MS2	HMDB	KEGG	Annotated
negative	10613	803	5843	5296	7066
positive	16776	1134	11735	10612	13194

Supplementary Table 29 Statistics for differential metabolites between different group comparisons.

Sample	All	Up	Down
3 dpi/CK	347	138	209
5 dpi/CK	210	116	94
7 dpi/CK	237	142	95
9 dpi/CK	266	168	98

Supplementary Table 30 KEGG enrichment result for differentially accumulated metabolites (top 20 pathways) in *P. sibirica* at 3 dpi.

KEGG A class	KEGG B class	Pathway	Count (75)	Pathway ID
Metabolism	Lipid metabolism	Glycerolipid metabolism	13	ko00561
Metabolism	Metabolism of cofactors and vitamins	Pantothenate and CoA biosynthesis	4	ko00770
Metabolism	Glycan biosynthesis and metabolism	Glycosylphosphatidylinositol (GPI)-anchor biosynthesis	2	ko00563
Environmental Information Processing	Signal transduction	Plant hormone signal transduction	2	ko04075
Metabolism	Biosynthesis of other secondary metabolites	Flavone and flavonol biosynthesis	4	ko00944
Metabolism	Lipid metabolism	Fatty acid biosynthesis	4	ko00061
Metabolism	Lipid metabolism	Arachidonic acid metabolism	5	ko00590
Metabolism	Lipid metabolism	Glycerophospholipid metabolism	6	ko00564
Metabolism	Nucleotide metabolism	Purine metabolism	9	ko00230
Metabolism	Lipid metabolism	alpha-Linolenic acid metabolism	5	ko00592
Metabolism	Amino acid metabolism	Valine, leucine and isoleucine biosynthesis	2	ko00290
Metabolism	Chemical structure transformation maps	Biosynthesis of plant secondary metabolites	6	ko01060
Metabolism	Global and overview maps	Metabolic pathways	51	ko01100
Metabolism	Biosynthesis of other secondary metabolites	Benzoxazinoid biosynthesis	1	ko00402
Metabolism	Lipid metabolism	Cutin, suberine and wax biosynthesis	2	ko00073
Metabolism	Lipid metabolism	Biosynthesis of unsaturated fatty acids	3	ko01040
Metabolism	Metabolism of other amino acids	beta-Alanine metabolism	2	ko00410
Metabolism	Biosynthesis of other secondary metabolites	Flavonoid biosynthesis	3	ko00941
Metabolism	Metabolism of other amino acids	Cyanoamino acid metabolism	2	ko00460
Metabolism	Energy metabolism	Nitrogen metabolism	1	ko00910

Supplementary Table 31 KEGG enrichment result for differentially accumulated metabolites (top 20 pathways) in *P. sibirica* at 5 dpi.

KEGG A class	KEGG B class	Pathway	Count (51)	Pathway ID
Metabolism	Glycan biosynthesis and metabolism	Glycosylphosphatidylinositol (GPI)-anchor biosynthesis	1	ko00563
Metabolism	Biosynthesis of other secondary metabolites	Flavonoid biosynthesis	4	ko00941
Metabolism	Chemical structure transformation maps	Biosynthesis of phenylpropanoids	6	ko01061
Metabolism	Lipid metabolism	alpha-Linolenic acid metabolism	3	ko00592
Metabolism	Carbohydrate metabolism	Galactose metabolism	3	ko00052
Metabolism	Lipid metabolism	Glycerophospholipid metabolism	3	ko00564
Metabolism	Global and overview maps	Metabolic pathways	37	ko01100
Metabolism	Nucleotide metabolism	Purine metabolism	5	ko00230
Metabolism	Metabolism of cofactors and vitamins	Pantothenate and CoA biosynthesis	2	ko00770
Environmental Information Processing	Signal transduction	Phosphatidylinositol signaling system	2	ko04070
Metabolism	Metabolism of other amino acids	beta-Alanine metabolism	2	ko00410
Metabolism	Nucleotide metabolism	Pyrimidine metabolism	4	ko00240
Metabolism	Lipid metabolism	Glycerolipid metabolism	7	ko00561
Environmental Information Processing	Signal transduction	Plant hormone signal transduction	1	ko04075
Metabolism	Carbohydrate metabolism	Inositol phosphate metabolism	2	ko00562
Metabolism	Amino acid metabolism	Histidine metabolism	3	ko00340
Metabolism	Biosynthesis of other secondary metabolites	Flavone and flavonol biosynthesis	2	ko00944
Metabolism	Amino acid metabolism	Glycine, serine and threonine metabolism	2	ko00260
Metabolism	Chemical structure transformation maps	Biosynthesis of plant secondary metabolites	4	ko01060
Metabolism	Lipid metabolism	Biosynthesis of unsaturated fatty acids	2	ko01040

Supplementary Table 32 KEGG enrichment result for differentially accumulated metabolites (top 20 pathways) in *P. sibirica* at 7 dpi.

KEGG_A_class	KEGG_B_class	Pathway	Count (70)	Pathway ID
Environmental Information Processing	Membrane transport	ABC transporters	9	ko02010
Metabolism	Biosynthesis of other secondary metabolites	Phenylpropanoid biosynthesis	6	ko00940
Metabolism	Lipid metabolism	alpha-Linolenic acid metabolism	7	ko00592
Metabolism	Amino acid metabolism	Arginine biosynthesis	3	ko00220
Metabolism	Chemical structure transformation maps	Biosynthesis of phenylpropanoids	6	ko01061
Metabolism	Chemical structure transformation maps	Biosynthesis of plant secondary metabolites	9	ko01060
Metabolism	Amino acid metabolism	Alanine, aspartate and glutamate metabolism	3	ko00250
Environmental Information Processing	Signal transduction	Plant hormone signal transduction	4	ko04075
Genetic Information Processing	Translation	Aminoacyl-tRNA biosynthesis	4	ko00970
Metabolism	Lipid metabolism	Glycerophospholipid metabolism	8	ko00564
Metabolism	Glycan biosynthesis and metabolism	Glycosylphosphatidylinositol (GPI)-anchor biosynthesis	3	ko00563
Metabolism	Metabolism of other amino acids	beta-Alanine metabolism	3	ko00410
Metabolism	Global and overview maps	Biosynthesis of amino acids	6	ko01230
Metabolism	Chemical structure transformation maps	Biosynthesis of plant hormones	6	ko01070
Metabolism	Amino acid metabolism	Histidine metabolism	4	ko00340
Metabolism	Metabolism of cofactors and vitamins	Nicotinate and nicotinamide metabolism	5	ko00760
Metabolism	Global and overview maps	Metabolic pathways	48	ko01100
Metabolism	Metabolism of cofactors and vitamins	Pantothenate and CoA biosynthesis	2	ko00770
Metabolism	Nucleotide metabolism	Purine metabolism	7	ko00230
Environmental Information Processing	Signal transduction	Phosphatidylinositol signaling system	4	ko04070

Supplementary Table 33 KEGG enrichment result for differentially accumulated metabolites (top 20 pathways) in *P. sibirica* at 9 dpi.

KEGG_A_class	KEGG_B_class	Pathway	Count (85)	Pathway ID
Metabolism	Biosynthesis of other secondary metabolites	Flavonoid biosynthesis	8	ko00941
Environmental Information Processing	Membrane transport	ABC transporters	10	ko02010
Metabolism	Amino acid metabolism	Arginine biosynthesis	5	ko00220
Metabolism	Chemical structure transformation maps	Biosynthesis of phenylpropanoids	10	ko01061
Metabolism	Amino acid metabolism	Alanine, aspartate and glutamate metabolism	5	ko00250
Metabolism	Biosynthesis of other secondary metabolites	Flavone and flavonol biosynthesis	5	ko00944
Genetic Information Processing	Translation	Aminoacyl-tRNA biosynthesis	6	ko00970
Metabolism	Chemical structure transformation maps	Biosynthesis of plant secondary metabolites	9	ko01060
Metabolism	Global and overview maps	Metabolic pathways	62	ko01100
Metabolism	Lipid metabolism	alpha-Linolenic acid metabolism	4	ko00592
Metabolism	Chemical structure transformation maps	Biosynthesis of alkaloids derived from ornithine, lysine and nicotinic acid	5	ko01064
Metabolism	Global and overview maps	Biosynthesis of amino acids	8	ko01230
Metabolism	Amino acid metabolism	Histidine metabolism	5	ko00340
Metabolism	Glycan biosynthesis and metabolism	Glycosylphosphatidylinositol (GPI)-anchor biosynthesis	3	ko00563
Metabolism	Nucleotide metabolism	Purine metabolism	9	ko00230
Metabolism	Energy metabolism	Nitrogen metabolism	3	ko00910
Metabolism	Carbohydrate metabolism	Butanoate metabolism	3	ko00650
Metabolism	Global and overview maps	2-Oxocarboxylic acid metabolism	6	ko01210
Metabolism	Carbohydrate metabolism	Galactose metabolism	3	ko00052
Metabolism	Metabolism of other amino acids	Cyanoamino acid metabolism	3	ko00460
